# Evaluation of the Antioxidant, Antimicrobial, and Anti-Biofilm Effects of the Stem Bark, Leaf, and Seed Extracts from *Hymenaea courbaril* and Characterization by UPLC-ESI-QTOF-MS/MS Analysis

**DOI:** 10.3390/antibiotics12111601

**Published:** 2023-11-08

**Authors:** Jhonatas Emílio Ribeiro da Cruz, Hellyssa Cataryna Saldanha, Andressa Moreira do Nascimento, Rafaela Barbosa Borges, Marcos de Souza Gomes, Guilherme Ramos Oliveira e Freitas, Carla Monteiro Leal, Everton Allan Ferreira, Ademar Alves da Silva Filho, Enyara Rezende Morais

**Affiliations:** 1 Institute of Biotechnology, Federal University of Uberlândia, Campus Patos de Minas, Rua Major Jerônimo, 566, sala 205, Patos de Minas 38700-002, MG, Brazil; hellyssa.saldanha@ufu.br (H.C.S.); andressa.moreira@ufu.br (A.M.d.N.); rafaela.barbosa@ufu.br (R.B.B.); grofreitas@ufu.br (G.R.O.e.F.); ermorais@ufu.br (E.R.M.); 2 Institute of Chemistry, Federal University of Uberlândia, Campus Patos de Minas, Rua Major Jerônimo, 566, Patos de Minas 38700-002, MG, Brazil; marcos.gomes@ufu.br; 3 Faculty of Pharmacy, Department of Pharmaceutical Sciences, Federal University of Juiz de Fora, Rua José Lourenço Kelmer, s/n, Juiz de Fora 36036-900, MG, Brazil; carla.leal@estudante.ufjf.br (C.M.L.); allan.everton@estudante.ufjf.br (E.A.F.); ademar.alves@ufjf.br (A.A.d.S.F.)

**Keywords:** antibacterial potential, antioxidant activity, biofilm inhibition, *Hymenaea courbaril*, *Staphylococcus aureus*

## Abstract

Currently, biofilm-forming bacteria are difficult to treat by conventional antibiotic therapy and are, thus, becoming a clinical and epidemiological problem worldwide. Medicinal plants have been identified as novel alternative treatments due to their therapeutic and antimicrobial effects. In this context, the present study aimed to determine the total phenolic content, antioxidant capacity, and antimicrobial and anti-biofilm potential of nine extracts of *Hymenaea courbaril* (Fabaceae), popularly known as Jatobá. Furthermore, extracts that exhibited biofilm inhibitory activity against *S. aureus* (ATCC 25923) were selected for UPLC-HRMS/MS chemical analysis. Our results showed a high total phenolic content, mainly in the stem bark extract, and that the plant is rich in compounds with antioxidant activity. In the anti-biofilm analysis, leaf extracts stood out in comparison with chloramphenicol, with inhibition percentages of 78.29% and 78.85%, respectively. Through chemical analysis by UPLC-HRMS/MS, chrysoeriol-7-*O*-neohesperidoside, isorhamnetin-3-*O*-glucoside, and 3,7-di-*O*-methylquercetin were annotated for the first time in the leaves of *H. courbaril*. Therefore, these results showed the potential use of *H. courbaril* as an antioxidant and point to its use in antimicrobial therapy with an anti-biofilm effect.

## 1. Introduction

According to data from the World Health Organization (WHO), about 700,000 people die annually from infections by resistant bacteria, and this number is projected to increase to 10 million deaths per year in 2050 [1]. In view of the difficulty of treating infections caused by *Staphylococcus aureus*, which produces biofilms, the need for novel and effective therapies is urgent [2,3]. For this reason, the development of novel therapeutic strategies, such as the use of medicinal plants with antimicrobial activity with a focus on inhibition or eradication of biofilm, is of great interest.

Some plant species and their phytoconstituents are promising sources of antimicrobial agents [4]. Thus, studies on the anti-infective activity of medicinal plants have become a progressive trend. Among the 162 new antibiotics approved in the period from 1981 to 2019, 94 of them originated from natural products [5].

*Hymenaea courbaril* (Fabaceae) is a medicinal plant species popularly known in Brazil as Jatobá that has a long history of use by Indigenous tribes in the Amazon basin, including communities in the Caatinga and Cerrado [6].

Plants of the genus *Hymenaea* (Fabaceae) are used in traditional South American and Asian medicine to treat a multitude of disorders, such as cough, diarrhea, dysentery, intestinal colic, lung weakness, asthma, anemia, and sore throat, as well as for the treatment of kidney disease and illnesses related to viruses and bronchitis [7].

Previous studies on the medicinal properties of *H. courbaril* have revealed antimicrobial activity against Gram-positive [8] and Gram-negative bacteria [9] and dengue virus type 2 [10]. However, little research has focused on the pharmacological activity of the stem bark and leaves of *H. courbaril* [7].

In this article, we demonstrate, for the first time, the anti-biofilm activity of *H. courbaril* leaf extracts and the annotation of their chemical compounds by UPLC-HRMS/MS that may contribute to the inhibition of a biofilm-forming *S. aureus*, a pathogenic bacterium of medical importance. It is noteworthy that there is no record in the literature, thus far, of investigations into the anti-biofilm activity of this species.

## 2. Results

### 2.1. Quantitative Determination of the Total Phenolic (PT) Content

**Figure 1:**
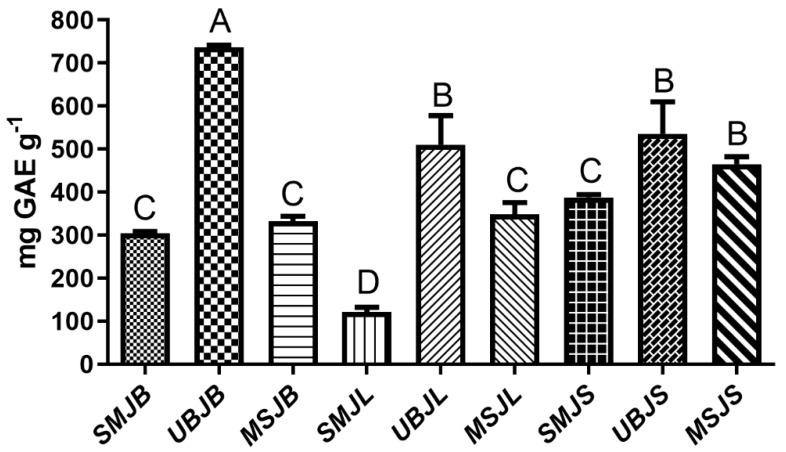
Total phenolic content of the crude extracts of Jatobá. Results are expressed as the mean ± standard deviation (n = 3). Means followed by the same letter in a column do not differ significantly at 5% probability by the Scott–Knott test. SMJB = Static Maceration Jatobá Bark; UBJB = Ultrasonic Bath Jatobá Bark; MSJB = Magnetic Stirring Jatobá Bark; SMJL = Static Maceration Jatobá Leaf; UBJL = Ultrasonic Bath Jatobá Leaf; MSJL = Magnetic Stirring Jatobá Leaf; SMJS = Static Maceration Jatobá Seed; UBJS = Ultrasonic Bath Jatobá Seed; MSJS = Magnetic Stirring Jatobá Seed.

The results offer new and valuable quantitative data on PT in *H. courbaril*, a plant popularly used in the Cerrado region, one of the areas with the greatest biodiversity in the world (Figure 1).

Bark extracts of Jatobá prepared by ultrasonic bath (UBJB; 736.1 mg GAE g^−1^) presented the highest levels of PT, followed by UBJS (535.7 mg GAE g^−1^) and, lastly, UBJL (510.6 mg GAE g^−1^), both statistically similar (Figure 1).

### 2.2. Determination of Antioxidant Activity

#### 2.2.1. Phosphomolybdenum Complex Reduction Method

Data on the total antioxidant activity of the nine Jatobá extracts determined by the phosphomolybdenum method expressed as milligrams of BHT Equivalent per gram of extract (mg BHTE g^−1^) are shown in Table 1.

It can be argued that the Jatobá leaf extracts showed a high level of total antioxidant capacity, with statistically similar values of 1213.0, 1195.5, and 1135.5 mg BHTE g^−1^ being obtained for the three extraction processes. On the other hand, seed (1030.0 mg BHTE g^−1^) and bark (1086.7 mg BHTE g^−1^) extracts showed statistically lower values (*p* < 0.05); this indicates that the leaves of this species can be considered an important source of compounds with antioxidant action, which makes them potentially useful for nutrition and pharmaceutical purposes.

**Figure 2:**
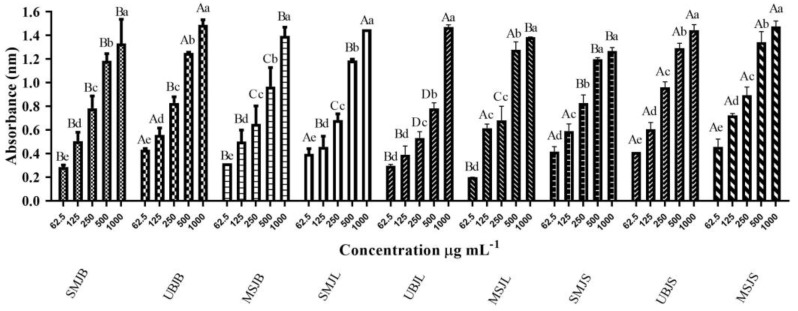
Reducing power of the phosphomolybdenum complex of the selected Jatobá extracts. Results expressed as the mean ± standard deviation (n = 3) at concentrations of 62.5–1000 µg mL^−1^. Means followed by the same letter do not differ significantly at 5% probability by the Scott–Knott test. Uppercase letters are used to compare the concentration between extracts, and lowercase to compare the concentration within each extract. SMJB = Static Maceration Jatobá Bark; UBJB = Ultrasonic Bath Jatobá Bark; MSJB = Magnetic Stirring Jatobá Bark; SMJL = Static Maceration Jatobá Leaf; UBJL = Ultrasonic Bath Jatobá Leaf; MSJL = Magnetic Stirring Jatobá Leaf; SMJS = Static Maceration Jatobá Seed; UBJS = Ultrasonic Bath Jatobá Seed; MSJS = Magnetic Stirring Jatobá Seed.

In Figure 2, it can be observed that the evaluated extracts demonstrated antioxidant activity, revealing a dependent relationship between absorbance (nm) and concentration. In other words, as the absorbance increases, the concentration of the extract also increases, with the increase in absorbance reflecting the increase in antioxidant activity.

#### 2.2.2. Ferric Reducing Antioxidant Power Assay

Table 2 shows data on the total antioxidant activity of the nine Jatobá extracts determined by the reducing power method, expressed in mg BHTE g^−1^.

Based on the above data, it was observed that the MSJL extract showed the greatest potential for reducing Fe^3+^, with a value of 834.3 mg BHTE g^−1^, followed by the UBJS extract (802.0 mg BHTE g^−1^) (*p* < 0.05). The values found showed that the leaf is an important plant organ for the storage of secondary metabolites with antioxidant potential. Jatobá leaves are known to possess important antioxidant constituents, including flavonoids, such as astilbin, and other phenolic compounds.

**Figure 3:**
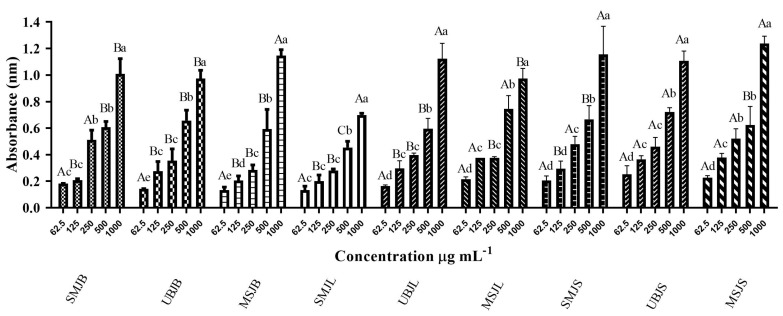
Ferric reducing antioxidant power (FRAP) of the selected Jatobá extracts. Results expressed as the mean ± standard deviation (n = 3) at concentrations of 62.5–1000 µg mL^−1^. Means followed by the same letter do not differ significantly at 5% probability by the Scott–Knott test. Uppercase letters are used to compare the concentration between extracts, and lowercase to compare the concentration within each extract. SMJB = Static Maceration Jatobá Bark; UBJB = Ultrasonic Bath Jatobá Bark; MSJB = Magnetic Stirring Jatobá Bark; SMJL = Static Maceration Jatobá Leaf; UBJL = Ultrasonic Bath Jatobá Leaf; MSJL = Magnetic Stirring Jatobá Leaf; SMJS = Static Maceration Jatobá Seed; UBJS = Ultrasonic Bath Jatobá Seed; MSJS = Magnetic Stirring Jatobá Seed.

The findings showed that absorbance values (nm) increased slightly for concentrations ranging from 62.5 to 500 µg mL^−1^ and then increased rapidly at a concentration of 1000 µg mL^−1^ (Figure 3).

### 2.3. Test for Susceptibility to the Antimicrobial Action of Plant Extracts

#### 2.3.1. Disc Diffusion Method

**Scheme 1:**
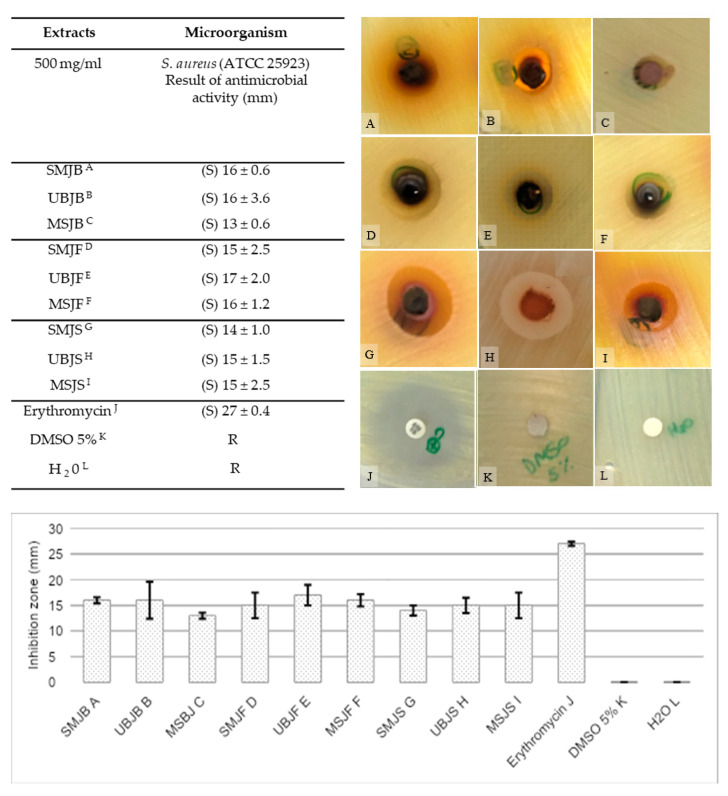
Top left: Mean of inhibition zones obtained from the disk diffusion tests in millimeters. S = Sensitive, R = Resistant. Data are shown as mean ± standard deviation from triplicate experiments. Top right: Inhibition halos. Letters indicate the correspondence between the extracts and controls and the pictures. Bottom: Graphic representation of the means. SMJB (**A**) = Static Maceration Jatobá Bark; UBJB (**B**) = Ultrasonic Bath Jatobá Bark; MSJB (**C**) = Magnetic Stirring Jatobá Bark; SMJL (**D**) = Static Maceration Jatobá Leaf; UBJL (**E**) = Ultrasonic Bath Jatobá Leaf; MSJL (**F**) = Magnetic Stirring Jatobá Leaf; SMJS (**G**) = Static Maceration Jatobá Seed; UBJS (**H**) = Ultrasonic Bath Jatobá Seed; MSJS (**I**) = Magnetic Stirring Jatobá Seed; (**J**) = Positive control for *S. aureus*; (**K**,**L**) = negative controls.

To assess antibacterial activity, the nine Jatobá extracts were tested at a concentration of 500 mg mL^−1^ against the Gram-positive species *S. aureus* (ATCC 25923) (Scheme 1).

#### 2.3.2. Broth Microdilution Method

The results of the anti-staphylococcal activity of Jatobá extracts are shown in Table 3. The results presented in this technique showed that the leaf extracts SMJL, UBJL, and MSJL, with MIC of 1.56, 1.56, and 3.12 mg mL^−1^, respectively, are better inhibitors than bark extracts (SMJB, UBJB, and MSJB), with MIC of 12.5 mg mL^−1^; this indicates the potential of *H. courbaril* leaves as a potent antibacterial agent.

On the other hand, Jatobá extracts from seeds (SMJS, UBJS, and MSJS) did not show antimicrobial activity against *S. aureus* (ATCC 25923); this can be explained by the fact that the solvent used (96% ethanol) was not able to adequately extract the bioactive compounds from the Jatobá seeds or, better yet, because the compounds of the Jatobá seeds are inactive or unable to penetrate the natural barrier of the cell wall of the bacterium *S. aureus*.

#### 2.3.3. Anti-Biofilm Activity against *S. aureus*

**Figure 4:**
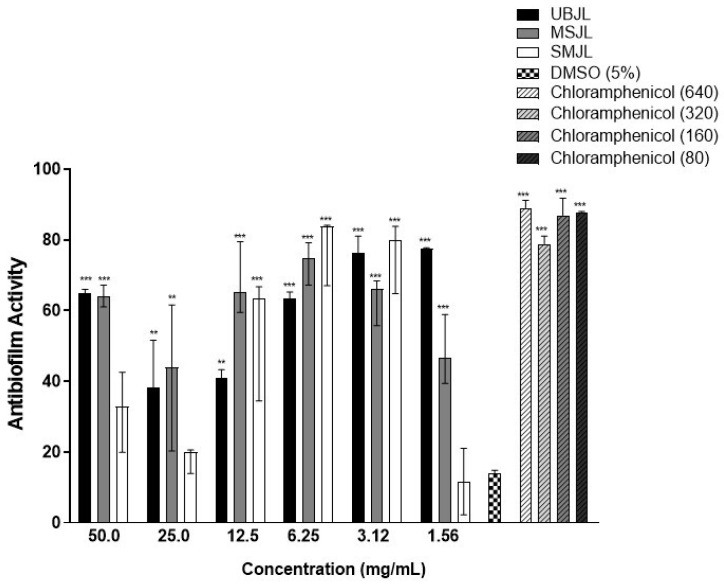
Activity of different concentrations of Jatobá leaf extracts against *S. aureus* biofilm (ATCC 25923). Anti-biofilm activity was calculated as a percentage by comparing the absorbance results (A560) of treated cells with those of untreated cells. The average values for treatment with each extract were compared to the average value for treatment with 5% DMSO (negative control). ** *p* < 0.01; *** *p* < 0.001). UBJL = Ultrasonic Bath Jatobá Leaf; MSJL = Magnetic Stirring Jatobá Leaf; SMJL = Static Maceration Jatobá Leaf.

With the results of the MIC test, the anti-biofilm activity of the most promising extracts (SMJL, UBJL, and MSJL) that showed antibacterial activity with an MIC equal to or less than 3.12 mg mL^−1^ for *S. aureus* (ATCC 25923) was determined (Figure 4). Different concentrations of the leaf extracts (50 to 1.56 mg mL^−1^) were prepared; they were inoculated on biofilms of *S. aureus*, and their activity was evaluated through the colorimetric MTT assay.

The three Jatobá leaf extracts (UBJL, MSJL, and SMJL) exhibited good results of biofilm inhibitory activity against *S. aureus*. Among the extracts tested, SMJL, at a concentration of 6.25 mg mL^−1^, showed the greatest anti-biofilm activity (78.29%) against *S. aureus*. This result is noteworthy since this in vitro anti-biofilm effect was statistically similar to that of chloramphenicol (inhibitory effect of 78.85%) (*p* < 0.001), which is the standard antimicrobial. Furthermore, it should be noted that no previous studies on the anti-biofilm activities of *H. courbaril* (Jatobá) leaf extracts against *S. aureus* have been reported. Thus, our findings are the first of their kind to be reported in the literature.

### 2.4. UPLC-HRMS/MS Analysis of H. courbaril Extracts

In this study, as the Jatobá leaf extracts (SMJL, UBJL, and MSJL) exhibited good results of biofilm inhibitory activity against *S. aureus*, they were selected for chemical profile analysis. Through UPLC-HRMS/MS analysis of SMJL, UBJL, and MSJL extracts, it was possible to obtain detailed chemical profiles and annotate many compounds in *H. courbaril*, especially flavonoids (Table 4) (see Supplementary Material).

**Figure 5:**
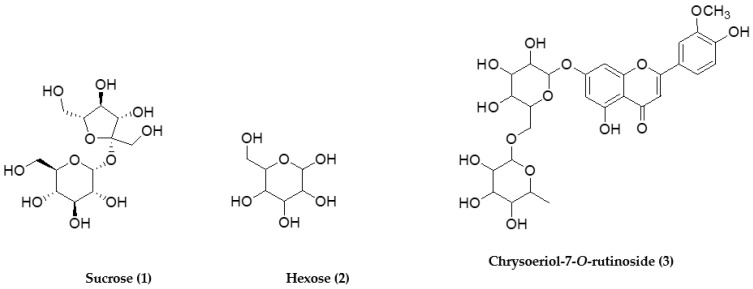

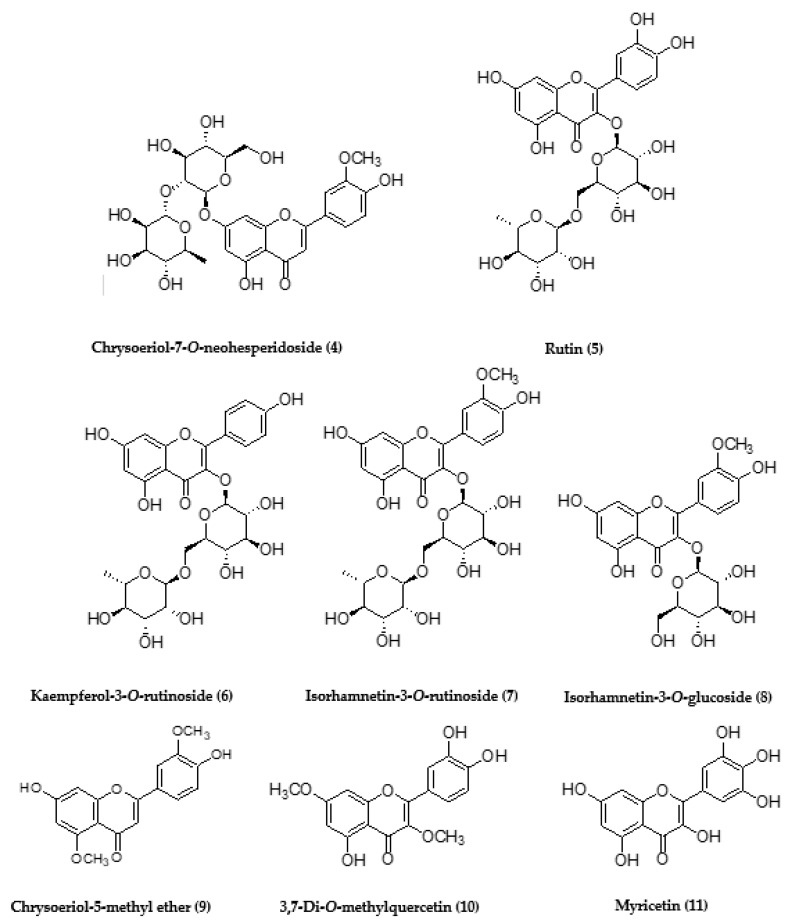

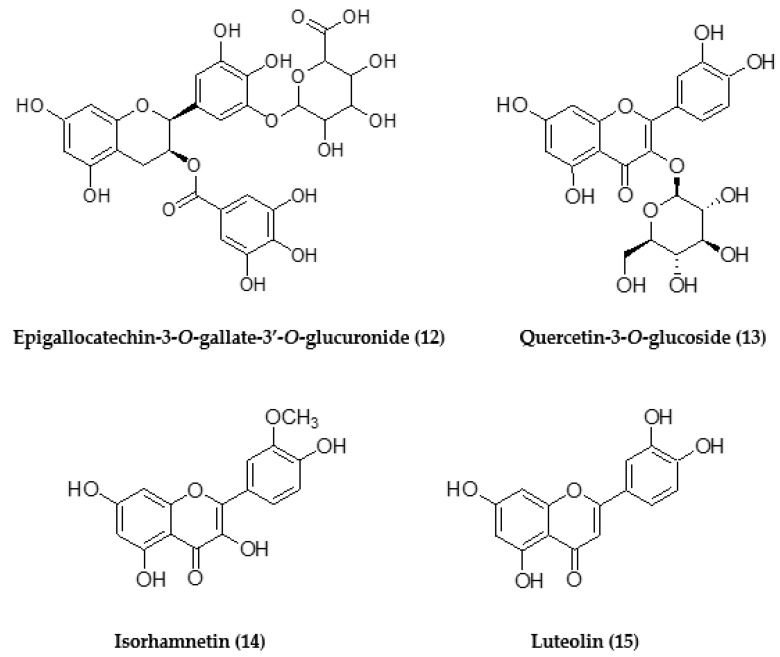
Annotated compounds in *H. courbaril* leaf extracts.

Our findings describe, for the first time, the annotation of the compounds chrysoeriol-7-*O*-neohesperidoside (**4**), isorhamnetin-3-*O*-glucoside (**8**), 3,7-di-*O*-methylquercetin (**10**), and myricetin (**11**) in the leaves of *H. courbaril* (Figure 5).

## 3. Discussion

The antioxidant, antimicrobial, and anti-biofilm properties of different *H. courbaril* extracts were investigated in our work, and the extracts that exhibited good biofilm inhibitory activity against *S. aureus* were also selected for UPLC-HRMS/MS chemical analyses.

Phenolic compounds are reported to have important therapeutic activity, such as antitumor action, and they may also act in the prevention of illnesses, such as cardiovascular disease, Alzheimer’s, Parkinson’s, cataracts, and diabetes [11,12,13]. Also, phenolic compounds are highly effective scavengers of most oxidizing molecules, including singlet oxygen and various free radicals implicated in diverse diseases [14,15]. Thus, phenolics are an important class of natural products with possible antioxidant action.

According to our results, a significant amount of phenolic content was detected in Jatobá extracts. Currently, special attention is given to natural products with an antioxidant function that may reduce oxidative stress and may be used in the treatment of infectious and/or inflammatory diseases [16].

Previous studies conducted with other plant species have shown a positive correlation between plant phenolic content and antioxidant properties [14,15], suggesting that the antioxidant action may be related to the phenolic content. Phenols may act as antioxidants not only because of their ability to donate hydrogen or electrons and chelating metals but also because of their stable intermediate radicals, which prevent oxidation [14].

Our findings demonstrated that at the lowest tested concentrations, all Jatobá extracts have free radical-scavenging metabolites with antioxidant activity, which are also correlated with the PT content in the extracts. Previous studies have shown that other *Hymenaea* species have interesting antioxidant capacity. In addition, Maranhão et al. [17] showed that quercetin, a flavonoid identified in *H. stignocarpa* heartwood extract, revealed promising antioxidant effects.

Our findings demonstrated that all Jatobá extracts exhibited high antioxidant capacity, except for seed extract, which presented weaker antioxidant properties in comparison with those from the leaves and bark. The leaves and stem bark, due to greater external environmental influences, may have a high content of phenolic compounds, which may confer selective advantages and defense mechanisms against microbial attacks, insect predation, and herbivorous animals [18].

Also, it was observed that the efficiency of extracting antioxidant compounds from the leaves was in the order of MS > UB > SM. Therefore, magnetic stirring, which promotes a homogeneous mixture and faster extraction [19], was more efficient for extracting the antioxidants present in Jatobá leaves. Another advantage of magnetic stirring is the use of room temperature, which may prevent the degradation of thermolabile metabolites [19]. However, the ultrasonic bath was more efficient for extracting antioxidant compounds from bark. As bark has high mechanical resistance, the ultrasonic bath causes cavitations and mechanical stress on the bark cells, increasing the capacity for mass transfer and cell rupture [20], which may improve the extraction of compounds. Therefore, the efficiency of the method used for extracting antioxidant compounds depends on which sample is used. If leaves are used, magnetic stirring is the best method, and if bark or seed are used, an ultrasonic bath guarantees better extraction.

Thus, in this context, Jatobá extracts are potentially effective in combating free radicals in humans, which are said to be involved in the genesis or development of various diseases via oxidative stress [15].

In addition, species of the genus *Hymenaea*, including *H. courbaril*, are popularly used in phytotherapy for the treatment of bacterial infections [6,7,21]. Therefore, the antibacterial activity of Jatobá extracts against *S. aureus* was also investigated. Our results showed that the leaf and bark extracts were active.

According to Chavasco et al. [22], plant products with antimicrobial activity at an MIC of less than 100 mg mL^−1^ are considered relevant for therapeutic applications. Thus, our antibacterial results showed the antibacterial potential of both Jatobá bark and leaf extracts, which exhibited action against *S. aureus* at concentrations lower than 100 mg mL^−1^. Interestingly, the Jatobá bark extracts SMJB, and UBJB showed antibiotic action against the *S. aureus* strain (ATCC 25923). These results are in agreement with those of Fernandes et al. [23], who reported the antibacterial properties of the hydroethanolic extract of the inner bark of *H. courbaril* against *S. aureus* (ATCC 10495).

Nevertheless, as the best antibacterial activity in the MIC test was found for the Jatobá leaf extracts, these were assayed in the anti-biofilm capacity of *S. aureus* since microbial biofilms increase bacterial resistance to various antimicrobial agents [2,3]. In this regard, investigation of the anti-biofilm effects of bioactive natural substances, such as Jatobá extracts, remains of interest. Also, no previous studies on the anti-biofilm activities of *H. courbaril* leaf extracts against *S. aureus* have been reported.

In the anti-biofilm capacity assay, all Jatobá leaf extracts presented good results of biofilm inhibitory activity against *S. aureus*. Among them, the SMJL extract showed the best anti-biofilm activity, with an anti-biofilm effect statistically comparable to that of chloramphenicol.

The antibacterial activity of *H. courbaril* leaf extracts against the *S. aureus* strain can be correlated with their chemical composition. Previous studies of this plant showed the presence of the flavonoid fisetin as the major compound that showed recognized antimicrobial properties [6]. In *H. courbaril*, there is a wide variety of antimicrobial agents, including phenolic and flavonoid compounds. Catechin and dihydrokaempferol-glucoside have been reported in the bark, while astilbin and epicatechin are found in the leaves of this plant [7]. Flavonoids are one of the main compounds responsible for antimicrobial activity, whose mechanism of action may be due to their ability to form complexes with the bacterial cell wall [24].

Our chemical characterization of the active Jatobá leaf extracts (SMJL, UBJL, and MSJL) by UPLC-HRMS/MS analysis showed detailed chemical profiles and the annotation of many compounds, especially phenolics and flavonoids, such as luteolin, rutin, and myricetin.

Some of the annotated compounds in the Jatobá leaf extracts have been previously reported in the genus *Hymenaea*, such as epigallocatechin-3-*O*-gallate-3′-*O*-glucuronide (**12**) [25] and luteolin (**15**), which were isolated from the leaves of *H. palustris* and demonstrated inhibitory activity against Gram-negative pathogens, such as *Neisseria gonorrhoeae* [26]. On the other hand, we are describing, for the first time, the annotation of the compounds chrysoeriol-7-*O*-neohesperidoside (**4**), isorhamnetin-3-*O*-glucoside (**8**), 3,7-di-*O*-methylquercetin (**10**), and myricetin (**11**) in the leaves of *H. courbaril*.

Also, previous studies have shown that some of the flavonoids that were annotated in *H. courbaril* extracts display promising antimicrobial properties that may contribute to the antibacterial activity of *H. courbaril* extracts against *S. aureus* biofilm, such as rutin (**5**) [27,28], myricetin (**11**) [29], and luteolin (**15**) [30].

Furthermore, our phytochemical investigation revealed that phenolic compounds were present at high concentrations in the leaf extracts. This observation may explain the anti-biofilm activity against the *S. aureus* strain since phenols are one of the main plant metabolites with good antimicrobial properties [16]. In this sense, the mechanisms of phenolic activity against microorganisms may include enzymatic inhibition and nonspecific interactions with membrane proteins [31]. However, as plant extracts comprise many components, it is expected that their mechanisms of action may involve multiple targets in bacterial cells rather than a single mechanism [32]. Thus, in our study, we believe that the bactericidal action against *S. aureus* may also be due to a synergism among all compounds in the extracts, especially phenolics.

Therefore, our results suggest that the phenolic compounds found in Jatobá extracts, especially flavonoids, are related to their antibacterial activity against *S. aureus*, although the effects of other possible active metabolites in *H. courbaril* cannot be discarded.

## 4. Material and Methods

### 4.1. Collection and Identification of Plant Material

The leaves, bark, and seeds of *H. courbaril* were collected on 14 December, 2020, at Rua Feliciana Donato de Macedo, Maravilha, Uberlândia, MG, 38401-844, Brazil (18°53′11.9″ S, 48°18′20.1″ W). The plant materials were identified by the agronomist, Prof. Dr. Terezinha Aparecida Teixeira. The specimen was registered and deposited in the Herbarium of the Federal University of Uberlândia (UFU) under the number HUFU 81526.

### 4.2. Preparation of Powdered Plant Material

Leaves, bark, and seeds of *H. courbaril* were first washed in water to remove impurities, and then all materials were stored separately in an ultra-freezer (Coldilab^®^, Piracicaba, SP, Brazil) at a temperature of −80 °C. In order to obtain crude extracts, plant materials were removed from the ultra-freezer and quickly placed to dry in a lyophilizer (Liotop^®^ L101, São Carlos, SP, Brazil) for a period of 72 h, followed by crushing in a mill with knives (Willey^®^ Star FT50, Piracicaba, SP, Brazil) into a fine powdered state.

### 4.3. Preparation of H. courbaril Extracts

#### 4.3.1. Extracts Obtained by Ultrasonic Bath Extraction

Powdered bark (4.95 g), leaves (4.86 g), and seeds (4.91 g) of Jatobá (*H. courbaril*) were separately submitted to extraction in an ultrasonic bath (Sanders Medical^®^—SoniClean 6, Santa Rita do Sapucaí, MG, Brazil), using 100 mL of 96% ethanol (Sigma^®^, St. Louis, MO, USA) as a solvent [33], at a fixed temperature of 40 °C, for 15 min, performed in three cycles. After filtration, the solvent was removed via rotary evaporation (Fisatom^®^, Perdizes, SP, Brazil) at 50 °C to yield crude extracts of Jatobá bark (UBJB, 3.36 g), leaves (UBJL, 3.59 g), and seeds (UBJS, 2.97 g). Finally, the crude extracts UBJB, UBJL, and UBJS were lyophilized, placed in 50-mL Falcon tubes, and stored in a refrigerator (4 °C).

#### 4.3.2. Extracts Obtained by Magnetic Stirring

Powdered bark (4.80 g), leaves (4.92 g), and seeds (4.87 g) of Jatobá (*H. courbaril*) were placed separately in 500-mL amber bottles, and 100 mL of 96% ethanol (Sigma^®^, St. Louis, MO, USA) was added, followed by constant stirring on a magnetic stirrer (Marconi^®^ MA–085L, Piracicaba, SP, Brazil) at 1200 rpm, protected from light, at room temperature (25 °C), for 24 h. Immediately after, the extracts were filtered separately under vacuum and dried on a rotary evaporator (Fisatom^®^, Perdizes, SP, Brazil) at 50 °C. At the end of rotary evaporation, to remove residual water, Jatobá bark (MSJB, 2.95 g), leaf (MSJL, 3.75 g), and seed (MSJS, 3.24 g) extracts obtained by magnetic stirring were placed in a lyophilizer (Liotop^®^ L101, São Carlos, SP, Brazil) for 24 h. The final preparations were placed in 50-mL Falcon tubes and stored in a refrigerator (4 °C).

#### 4.3.3. Extracts Obtained by Static Maceration

Powdered bark (4.95 g), leaves (4.93 g), and seeds (4.90 g) of Jatobá (*H. courbaril*) were placed separately in 500-mL amber bottles, and then 100 mL of 96% ethanol (Sigma^®^, St. Louis, MO, USA) was added and the bottles left to rest, protected from light, at room temperature (25 °C), for 24 h. This procedure was repeated three times for maximum extraction (depletion) of all compounds by the solvent. Then, the extracts were vacuum-filtered and dried in a rotary evaporator (Fisatom^®^, Perdizes, SP, Brazil) at 50 °C. At the end of rotary evaporation, to remove the remaining water, Jatobá bark (SMJB, 3.01 g), leaf (SMJL, 3.62 g), and seed (SMJS, 3.40 g) extracts obtained by static maceration were lyophilized (Liotop^®^ L101, São Carlos, SP, Brazil) for 24 h. The final preparations were placed in 50-mL Falcon tubes and stored in a refrigerator (4 °C).

### 4.4. Determination of PT Content

An aliquot of 0.5 mL of the diluted sample (50 g L^−1^) of each extract of Jatobá bark (UBJB, MSJB, SMJB), leaves (UBJL, MSJL, SMJL), and seeds (UBJS, MSJS, SMJS) was mixed with 2.5 mL of Folin–Ciocalteu reagent (Sigma; 0.2 mol L^−1^) [34]. Subsequently, 2 mL of saturated sodium carbonate solution (Sigma; 75 g L^−1^) was added to the reaction mixture. Absorbance readings were taken at 760 nm after incubation at room temperature for 2 h. Gallic acid (Neon^®^, Suzano, SP, Brazil) was used as a reference standard, and the results were expressed in milligrams of gallic acid equivalent (mg GAE) per gram of dry weight of plant material. All tests were performed in triplicate.

### 4.5. Analysis of Antioxidant Activity

#### 4.5.1. Phosphomolybdenum Complex Reduction Method

To prepare the reaction medium, an aliquot of 100 µL of each extract of Jatobá bark (UBJB, MSJB, SMJB), leaves (UBJL, MSJL, SMJL), and seeds (UBJS, MSJS, SMJS) at a concentration of 100 µL mL^−1^ was conditioned in test tubes, and then 1 mL of the reagent (0.6 M sulfuric acid; 28 mM sodium phosphate, and 4 mM ammonium molybdate; Sigma) was added to the same tubes [35]. The tubes were capped and incubated in a water bath (Cientec^®^, Belo Horizonte, MG, Brazil) at 95 °C for 60 min. After the test tubes were cooled, readings were performed in a spectrophotometer (Gehaka^®^ UV-340G, São Paulo, SP, Brazil) at 695 nm. The values were compared with a standard curve of several concentrations of the synthetic antioxidant BHT (butylhydroxytoluene; Sigma) (1000, 500, 250, 125, and 62.5 μg mL^−1^). The results were expressed in milligrams of BHT Equivalent per gram of dry weight of plant material (mg BHTE g^−1^).

#### 4.5.2. Ferric Reducing Power Test

Antioxidant activity was evaluated through the method of reducing power according to the methodology of Oyaizu [36]. An aliquot of 100 µL of each extract of Jatobá bark (UBJB, MSJB, SMJB), leaves (UBJL, MSJL, SMJL), and seeds (UBJS, MSJS, SMJS) at a concentration of 100 µL mL^−1^ was conditioned in test tubes, and then 1 mL each of 0.2 M phosphate buffer pH 6.0 and 1% potassium ferrocyanide aqueous solution was added to the same tubes. After 20 min of incubation in a water bath at 50 °C (Cientec^®^), 1 mL of 10% trichloroacetic acid was added to the mixture. Then, 3 mL of distilled water and 600 µL of 0.1% ferric chloride were added. Readings were performed using a spectrophotometer (Gehaka^®^) at 700 nm. The values were compared with a curve of several concentrations of the synthetic antioxidant BHT (Sigma^®^) (1000, 500, 250, 125, and 62.5 μg mL^−1^). The results were expressed in milligrams of BHT Equivalent per gram of extract (mg BHTE g^−1^).

### 4.6. Evaluation of the Antimicrobial Activity of Extracts by Disk Diffusion in Agar

The microorganism used to screen for sensitivity to plant extracts was *S. aureus* (ATCC 25923). The disk diffusion method in agar was used according to the standards described by the Clinical and Laboratory Standards Institute [37]. Bacteria were kept on tryptone soy agar (TSA; Kasvi^®^, São Paulo, SP, Brazil), incubated for 24 h at 37 °C, resuspended in sterile 0.85% saline solution, and adjusted according to the 0.5 McFarland standard (10^8^ CFU mL^−1^).

The suspension containing the bacteria was homogeneously inoculated in Petri dishes containing 70 mL of Mueller–Hinton agar (MHA; bioMérieux^®^, Jacarepaguá, RJ, Brazil). After 15 min, sterile filter paper discs (6 mm in diameter) containing 10 μL of fractions diluted to 500 mg mL^−1^ were deposited on the surface of the culture medium, with a minimum distance of 24 mm between them, and the plates were then incubated at 37 °C for 24 h. The tests were performed in triplicate. For the experiment, 15 µg of the antibiotic erythromycin (Cecon^®^, São Paulo, SP, Brazil) was used as a positive control for *S. aureus,* and the compounds 96% ethanol (Sigma) and 5% DMSO (Sigma) were used as negative controls.

### 4.7. Determination of Minimum Inhibitory Concentration (MIC) by Broth Microdilution

The extracts of Jatobá bark (UBJB, MSJB, SMJB) and leaves (UBJL, MSJL, SMJL) that presented antibacterial activity in the preliminary assessment were subjected to MIC determination by the broth microdilution technique [37,38]. Tests were performed in Mueller–Hinton medium (MHB; bioMérieux) in a sterile 96-well plate. An aliquot of 10 μL of each plant extract of Jatobá bark (UBJB, MSJB, SMJB) and leaves (UBJL, MSJL, SMJL) at concentrations of 1.562, 3.125, 6.25, 12.5, 25 and 50 mg mL^−1^ was deposited in each well containing MHB and microorganism suspension to a final volume of 200 μL per well. For the experiment, 5% DMSO (Sigma) was added as a negative control and 30 mg mL^−1^ chloramphenicol (Cecon) as a positive control.

All plates were incubated at 37 °C for 24 h. To indicate bacterial growth, 10 µL of bromide in methylthiazolyldiphenyl tetrazolium (MTT; Sigma) at 0.2 mg mL^−1^ was added to each well and incubated for 4 h. The MIC was defined as the lowest fractional concentration able to inhibit microbial growth.

### 4.8. Biofilm Inhibition Assay Preformed In Vitro

The anti-biofilm assay was a minor modification of the method reported by Brambilla et al. [39]. Biofilms of *S. aureus* (ATCC 25923) were formed in 96-well polystyrene microtiter plates. A suspension in 100 µL that contained 10^8^ cells mL^−1^ in tryptone soy broth (TSB; Kasvi) was inoculated into the wells and incubated at 37 °C for 24 h under agitation at 120 rpm. The well contents were discharged, and the wells were washed with 0.9% saline. Dilutions of Jatobá leaf extracts (UBJL, MSJL, SMJL) at concentrations of 50, 25, 12.5, 6.25, 3.12, and 1.56 mg mL^−1^ and the standard drug chloramphenicol as a positive control (Cecon) at concentrations of 640, 320, 160, and 80 µL mL^−1^ were then added to each well. After incubation at 37 °C for 24 h, the wells were washed with 0.9% saline solution. The MTT reduction assay (Sigma) was performed to assess the viability of biofilms. MTT solution (50 µL; 2 mg mL^−1^; Sigma) was added to each well, and the plates were incubated at 37 °C for 2 h. After color development, the solution in MTT was removed from each well, and 100 µL of 5% DMSO (Sigma) was added to dissolve the MTT crystals (Sigma). Subsequently, 100 µL of the resulting-colored solution was transferred to a new plate, and the optical density was measured at 560 nm using a microplate reader (Multiskan™ FC, Thermo Scientific, Waltham, MA, USA). The assays were performed in triplicate.

The standard antibiotic (chloramphenicol at concentrations of 640, 320, 160, and 80 µg mL^−1^; Sigma) was used as a positive control for biofilm prevention, 5% DMSO (Sigma), TSB medium (Kasvi^®^), and half TSB (Kasvi) in wells without bacterial inoculum was used as the negative control, treatment control, and sterility control, respectively.

The absorbance obtained is inversely proportional to the anti-biofilm activity, which was calculated using the values of the wells treated with the different concentrations of the extracts and the values of wells to which only TSB medium (treatment control; Kasvi) was added, using the following formula:

$$AA\;\left(\%\right)=\left(1-\frac{DOext-DOb\;}{DOct-DOb}\right)\times 100$$

where:

AA (%) = anti-biofilm activity as a percentage;

DOext = average optical density of treatment with extracts at many different concentrations;

DOb = median optical density of wells without the addition of reagents (white);

DOct = average optical density of the control treatment.

### 4.9. UPLC-HRMS/MS Analysis

The leaf extracts of Jatobá obtained by the static maceration (SMJL), ultrasonic bath (UBJL), and magnetic stirring (MSJL) methods were analyzed by ultra-performance liquid chromatography (UPLC) using an ACQUITY UPLC system (Waters Corporation, Milford, MA, USA) equipped with a binary pump, in-line degasser, and autosampler coupled to an electrospray ionization quadrupole time-of-flight tandem mass spectrometer (ESI-QTOF-MS/MS) (Waters Corporation, Milford, MA, USA). The samples were dissolved in methanol (10 mg mL^−1^), centrifuged at 10,000 rpm, and filtered with a 0.22-μm filter, and 5 μL was injected at a flow rate of 0.4 mL min^−1^. Chromatographic analysis was performed on a BEH C18 column (100 mm × 2.1 mm, 1.7 μm; Milford, MA, USA), and the mobile phase consisted of 0.1% formic acid (A) and LC-grade acetonitrile (B) with the following gradient profiles: 0–2 min, 5% B; 2–14 min, 5%–98% B; 14–16 min, 98% B; and 16–20 min, 98–5% B. Mass spectrometry analysis was performed with a XEVO G2S QTOF mass spectrometer (Waters Corporation, Milford, MA, USA) equipped with an ESI ionization source, operated in negative and positive modes. The capillary voltage was 3.0 kV, the lowest collision energy was 6 eV, and the highest collision energy was 15 to 30 eV. The ion source temperature was 120 °C, and the desolvation temperature was 450 °C. Nitrogen was used as a source of desolvation gas (800 L h^−1^) and cone gas (50 L h^−1^). For accurate mass measurements, data were centered during acquisition and 200 pg mL^−1^ leucine-enkephalin (*m*/*z* 565.2771) (Sigma-Aldrich, Steinheim, Germany), dissolved in acetonitrile/0.1% formic acid (50:50, *v/v*), was continuously infused as an external reference (LockSpray™) into the ESI source with automatic mass correction enabled. The full scan acquisition was in the mass range *m*/*z* 100–1000, and the acquired data were processed using the ChromaLynx™ application manager with MassLynx™ 4.1 software (Waters Corporation, Milford, MA, USA). The MS^2^ spectra were compared with literature and online databases such as PubChem, ChemSpider, and MassBank.

### 4.10. Statistical Analysis

For the evaluation of PT content and antioxidant activity, the experiment was arranged in a completely randomized design for the nine types of extracts: bark (UBJB, MSJB, SMJB), leaves (UBJL, MSJL, SMJL), and seeds (UBJS, MSJS, SMJS), in three replicates. The statistical program used was the Analysis System of Variance for Balanced Data (Sisvar), and the data were submitted for analysis of variance and the means compared with a Scott–Knott test at the 5% probability level.

GraphPad Prism (v.5) software was used to analyze the results of the anti-biofilm test. Means and standard deviations were calculated for each of the treatments and compared with the results found for the group treated with 5% DMSO (negative control; Sigma). The distribution from dice was evaluated, and the comparison between groups fulfilled the assumptions of ANOVA and Tukey’s post hoc test for multiple comparisons. Differences were considered statistically significant at *p* < 0.05.

## 5. Conclusions

The prevalence of phenolic compounds observed in the *H. courbaril* extracts provides evidence that this plant is a potential source of metabolites with reducing power. The results of the phosphomolybdenum assay showed that Jatobá leaf extracts efficiently inhibited free radicals in vitro. Also, SMJL, UBJL, and MSJL extracts exhibited biofilm-forming inhibitory activity against *S. aureus* similar to standard drugs, indicating their potential to be used as anti-biofilm agents. Furthermore, our results suggest that some of the bioactive compounds annotated in *H. courbaril* leaf extracts by UPLC-HRMS/MS analysis, such as flavonoids, may contribute to the antibacterial activity against *S. aureus* biofilm. Moreover, our findings give support for the medicinal use of Jatobá in the treatment of infections caused by *S. aureus* and suggest that *H. courbaril* leaf extracts may represent an alternative to overcome the disturbing phenomenon of antimicrobial resistance in biofilms. Finally, even though *H. courbaril* leaf extracts may be considered promising natural samples against *S. aureus* biofilms; further clinical and antibacterial studies are needed.

## Figures and Tables

**Table 1:** Values of total antioxidant activity of Jatobá extracts by the phosphomolybdenum method.

Extracts	Phosphomolybdenum Complex mg BHTE g^−1^	A *p*-Value < 0.05 Was Considered Significant
SMJB	1086.7	(B)
UBJB	1231.0	(A)
MSJB	1218.2	(A)
SMJL	1195.5	(A)
UBJL	1213.4	(A)
MSJL	1135.5	(A)
SMJS	1030.0	(B)
UBJS	1190.3	(A)
MSJS	1218.2	(A)

Mean values followed by the same letter in a column do not differ significantly at 5% probability by the Scott–Knott test. Results are expressed as the mean ± standard deviation (n = 3). SMJB = Static Maceration Jatobá Bark; UBJB = Ultrasonic Bath Jatobá Bark; MSJB = Magnetic Stirring Jatobá Bark; SMJL = Static Maceration Jatobá Leaf; UBJL = Ultrasonic Bath Jatobá Leaf; MSJL = Magnetic Stirring Jatobá Leaf; SMJS = Static Maceration Jatobá Seed; UBJS = Ultrasonic Bath Jatobá Seed; MSJS = Magnetic Stirring Jatobá Seed.

**Table 2:** Values of total antioxidant activity of Jatobá extracts by the reducing power method.

Extracts	Reducing Power mg BHTE g^−1^	A *p*-Value < 0.05 Was Considered Significant
SMJB	636.7	(B)
UBJB	708.1	(B)
MSJB	617.2	(B)
SMJL	418.1	(B)
UBJL	620.0	(B)
MSJL	834.3	(A)
SMJS	721.0	(B)
UBJS	802.0	(A)
MSJS	659.6	(B)

Means followed by the same letter in a column do not differ significantly at 5% probability by the Scott–Knott test. Results are expressed as the mean ± standard deviation (n = 3). SMJB = Static Maceration Jatobá Bark; UBJB = Ultrasonic Bath Jatobá Bark; MSJB = Magnetic Stirring Jatobá Bark; SMJL = Static Maceration Jatobá Leaf; UBJL = Ultrasonic Bath Jatobá Leaf; MSJL = Magnetic Stirring Jatobá Leaf; SMJS = Static Maceration Jatobá Seed; UBJS = Ultrasonic Bath Jatobá Seed; MSJS = Magnetic Stirring Jatobá Seed.

**Table 3:** Minimum inhibitory concentration (MIC) values of plant extracts and antibiotic correspondence against a strain of *S. aureus*.

Extracts	Microorganism
	*S. aureus* (ATCC 25923) Minimum Inhibitory Concentration (mg/mL)
SMJB ^a^	12.5
UBJB ^b^	12.5
MSJB ^c^	12.5
SMJL ^d^	1.56
UBJL ^e^	1.56
MSJL ^f^	3.12
SMJS ^g^	S/A ^l^
UBJS ^h^	S/A
MSJS ^i^	S/A
Chloramphenicol ^j^	32.0 ± 0.4 µg/mL
DMSO 5% ^k^	S/A
H_2_ O ^k^	S/A

Data are shown as mean ± standard deviation from triplicate experiments. ^a^ SMJB = Static Maceration Jatobá Bark; ^b^ UBJB = Ultrasonic Bath Jatobá Bark; ^c^ MSJB = Magnetic Stirring Jatobá Bark; ^d^ SMJL = Static Maceration Jatobá Leaf; ^e^ UBJL = Ultrasonic Bath Jatobá Leaf; ^f^ MSJL = Magnetic Stirring Jatobá Leaf; ^g^ SMJS = Static Maceration Jatobá Seed; ^h^ UBJS = Ultrasonic Bath Jatobá Seed; ^i^ MSJS = Magnetic Stirring Jatobá Seed; ^j^ Positive control for *S. aureus*; ^k^ Negative control; ^l^ S/A: No activity.

**Table 4:** UPLC-HRMS/MS data of annotated compounds in *H. courbaril* extracts.

R*_t_* (min)	[M − H]^−^ (*m*/*z*)	[M + H]^+^ (*m*/*z*)	Molecular Formula	MS/MS (MS^2^)	Proposed Compounds
			SMJL ^a^		
0.60	341.0149	-	C_12_H_22_O_11_	179.0089	Sucrose (**1**)
0.60	179.0089	-	C_6_H_12_O_6_	161.0020, 149.0117, 142.9959, 131.0033, 119.0040, 100.9987, 89.0012, 70.9955	Hexose (**2**)
5.27	607.9508	-	C_28_H_32_O_15_	299.9359	Chrysoeriol-7-*O*-rutinoside (**3**) or Chrysoeriol-7-*O*-neohesperidoside (**4**)
5.28	609.9561	-	C_27_H_30_O_16_	301.9397	Rutin (**5**)
5.62	593.0129	-	C_27_H_30_O_15_	285.9650	Kaempferol-3-*O*-rutinoside (**6**)
5.68	623.0092	-	C_28_H_32_O_16_	315.9617	Isorhamnetin-3-*O*-rutinoside (**7**)
5.78	477.9869	-	C_22_H_22_O_12_	315.9658, 299.9429, 270.9479	Isorhamnetin-3-*O*-glucoside (**8**)
7.38	313.9497	-	C_17_H_14_O_6_	298.9301, 270.9445	Chrysoeriol-5-methyl ether (**9**)
7.38	329.9728	-	C_17_H_14_O_7_	314.9513, 299.9359	3,7-Di-*O*-methylquercetin (**10**)
8.37	317.1195	-	C_15_H_10_O_8_	289.1298	Myricetin (**11**)
14.19	633.3182	-	C_28_H_26_O_17_	305.1683	Epigallocatechin-3-*O*-gallate-3′-*O*- glucuronide (**12**)
5.37	-	633.7400 [M + Na]^+^	C_27_H_30_O_16_	465.5418, 303.3375	Rutin (**5**)
5.49	-	487.5446 [M + Na]^+^	C_21_H_20_O_12_	303.3339	Quercetin-3-*O*-glucoside (**13**)
5.74	-	317.3806	C_16_H_12_O_7_	302.3412	Isorhamnetin (**14**)
			UBJL ^b^		
0.60	341.0376	-	C_12_H_22_O_11_	179.0199	Sucrose (**1**)
0.60	179.0199	-	C_6_H_12_O_6_	161.0124, 149.0217, 143.0057, 131.0127, 119.0107, 101.0049, 89.0070, 71.0007	Hexose (**2**)
5.26	607.9912	-	C_28_H_32_O_15_	299.9536	Chrysoeriol-7-*O*-rutinoside (**3**) or Chrysoeriol-7-*O*-neohesperidoside (**4**)
5.26	609.9966	-	C_27_H_30_O_16_	301.9610	Rutin (**5**)
5.67	623.0296	-	C_28_H_32_O_16_	315.9726	Isorhamnetin-3-*O*-rutinoside (**7**)
6.71	285.9754	-	C_15_H_10_O_6_	150.9716, 132.9994	Luteolin (**15**)
7.38	313.9642	-	C_17_H_14_O_6_	298.9443, 270.9580	Chrysoeriol-5-methyl ether (**9**)
7.38	329.9876	-	C_17_H_14_O_7_	314.9695, 299.9500	3,7-Di-*O*-methylquercetin (**10**)
5.28	-	633.1422 [M + Na]^+^	C_27_H_30_O_16_	465.1071, 303.0519	Rutin (**5**)
5.40	-	487.0886 [M + Na]^+^	C_21_H_20_O_12_	303.0519	Quercetin-3-*O*-glucoside (**13**)
5.67	-	317.0632	C_16_H_12_O_7_	302.0388	Isorhamnetin (**14**)
			MSJL ^c^		
0.60	341.0451	-	C_12_H_22_O_11_	179.0253	Sucrose (**1**)
0.60	179.0253	-	C_6_H_12_O_6_	161.0176, 143.0105, 131.0150, 119.0151, 101.0069, 89.0090, 71.0024	Hexose (**2**)
5.28	609.0568	-	C_27_H_30_O_16_	301.9893	Rutin (**5**)
5.67	623.1573	-	C_28_H_32_O_16_	315.0493	Isorhamnetin-3-*O*-rutinoside (**7**)
7.39	329.0812	-	C_17_H_14_O_7_	314.0585, 299.0328	3,7-Di-*O*-methylquercetin (**10**)
14.18	633.5037	-	C_28_H_26_O_17_	305.2577	Epigallocatechin-3-*O*-gallate-3′-*O*- glucuronide (**12**)

^a^ SMJL = Static Maceration Jatobá Leaf; ^b^ UBJL = Ultrasonic Bath Jatobá Leaf; ^c^ MSJL = Magnetic Stirring Jatobá Leaf.

## Data Availability

The data presented in this study are available in the article and supplementary material.

## Supplementary Materials

The following supporting information can be downloaded at https://www.mdpi.com/article/10.3390/antibiotics12111601/s1, Figures S1–S31: LC-MS data of Jatoba extracts.

## References

[1] Shankar P., Palaian S. (2016). Book Review: Responsible Use of Medicines—A Layman′s Handbook. Arch. Pharm. Pract..

[2] Jara M.C., Frediani A.V., Zehetmeyer F.K., Bruhn F.R.P., Müller M.R., Miller R.G., Nascente P.D.S. (2021). Multidrug-Resistant Hospital Bacteria: Epidemiological Factors and Susceptibility Profile. Microb. Drug Resist..

[3] Schilcher K., Horswill A.R. (2020). Staphylococcal Biofilm Development: Structure, Regulation, and Treatment Strategies. Microbiol. Mol. Biol. Rev..

[4] Ben Abdallah F., Lagha R., Gaber A. (2020). Biofilm Inhibition and Eradication Properties of Medicinal Plant Essential Oils against Methicillin-Resistant *Staphylococcus aureus* Clinical Isolates. Pharmaceuticals.

[5] Newman D.J., Cragg G.M. (2020). Natural Products as Sources of New Drugs over the Nearly Four Decades from 01/1981 to 09/2019. J. Nat. Prod..

[6] Costa M.P., Bozinis M.C.V., Andrade W.M., Costa C.R., da Silva A.L., Alves de Oliveira C.M., Kato L., de Fernandes O.F.L., Souza L.K.H., do Silva M.R.R. (2014). Antifungal and Cytotoxicity Activities of the Fresh Xylem Sap of *Hymenaea courbaril* L. and Its Major Constituent Fisetin. BMC Complement. Altern. Med..

[7] Boniface P.K., Baptista Ferreira S., Roland Kaiser C. (2017). Current State of Knowledge on the Traditional Uses, Phytochemistry, and Pharmacology of the Genus Hymenaea. J. Ethnopharmacol..

[8] De Brito M.S., Melo M.B., Perdigão J., Alves D.A., Fontenelle S., Feliciano M., Betânia L. (2016). Partial Purification of Trypsin/Papain Inhibitors from *Hymenea courbaril* L. Seeds and Antibacterial Effect of Protein Fractions Materials and Methods. Hoehnea.

[9] Aleixo A.A., Camargos V.N., Herrera K.M.S., dos Santos Pereira Andrade A.C., dos Santos M., Miranda V.C., Carvalho R.S., de MagalhÃ£es J.T., de MagalhÃ£es J.C., dos Santos Lima L.A.R. (2015). Synergistic Activity Of *Hymenaea courbaril* Terra *Stryphnodendron adstringens* (Mart.) Coville Against Multidrug-resistant Bacterial Strains. J. Med. Plant Res..

[10] Zandi K., Teoh B.T., Sam S.S., Wong P.F., Mustafa M.R., Abubakar S. (2011). In Vitro Antiviral Activity of Fisetin, Rutin and Naringenin against Dengue Virus Type-2. J. Med. Plant Res..

[11] Rocha E.C., Sartori C.A., Navaro F.F. (2016). The Application of Antioxidant Foods in the Prevention of Skin Aging. Rev. Cient. Uniararas.

[12] Barroqueiro E.S.B., Prado D.S., Barcellos P.S., Silva T.A., Pereira W.S., Silva L.A., Maciel M.C.G., Barroqueiro R.B., Nascimento F.R.F., Gonçalves A.G. (2016). Immunomodulatory and Antimicrobial Activity of Babassu Mesocarp Improves the Survival in Lethal Sepsis. Evid. Based Complement. Altern. Med..

[13] Silva C.M., Santos R.A., Cavalcante C.F.E. (2016). The Benefits of Nutrition in Preventing Skin Aging. Rev. Conex. Eletron..

[14] Cruz J.E.R., Costa G.J.F., Souza G.M., Freitas G.R.O., Morais E.R. (2019). Phytochemical Analysis and Evaluation of Antimicrobial Activity of *Peumus boldus*, *Psidium guajava*, *Vernonia polysphaera*, *Persea americana*, *Eucalyptus citriodora* Leaf Extracts and *Jatropha multifida* Raw Sap. Curr. Pharm. Biotechnol..

[15] Alexandre G.P., Simão J.L.S., Tavares M.O.A., Zuffo I.M.S., Prado S.V., De Paiva J.A., Mustapha A.N., De Oliveira A.E., Kato L., Severino V.G.P. (2022). Dereplication by HPLC-ESI-MS and Antioxidant Activity of Phenolic Compounds from *Banisteriopsis laevifolia* (Malpighiaceae). An. Acad. Bras. Cienc..

[16] Lemos A.S.O., Campos L.M., Souza T.F., Granato J.T., Oliveira E.E., Aragão D.M.O., Apolônio A.C.M., Ferreira A.P., Fabri R.L. (2022). Combining UFLC-QTOF-MS Analysis with Biological Evaluation of *Centrosema coriaceum* (Fabaceae) Leaves. An. Acad. Bras. Cienc..

[17] Maranhão C.A., Pinheiro I.O., Santana A.L.B.D., Oliveira L.S., Nascimento M.S., Bieber L.W. (2013). Antitermitic and Antioxidant Activities of Heartwood Extracts and Main Flavonoids of *Hymenaea stigonocarpa* Mart. Int. Biodeterior. Biodegrad..

[18] Singh S., Kaur I., Kariyat R. (2021). The Multifunctional Roles of Polyphenols in Plant-Herbivore Interactions. Int. J. Mol. Sci..

[19] Cruz J.E.R., Ribeiro P.S., Farisco F., Gomes M.S., Morais E.R. (2022). Composition of Phenolics, Flavonoids, Anthocyanins, Determination of Antioxidant Activity and Comparison of Extractive Methods of *Caryocar brasiliense* Leaf and Bark. Rev. Ciênc. Méd. Biol..

[20] Sarker S.D., Latif Z., Gray A.I. (2007). Nactural Products Isolation.

[21] Cruz J.E.R., Guimarães I.I.S.M., Almeida K.C., Amâncio N.F.G. (2023). Antifungal and antibacterial activities of the medicinal plant Jatobá (*Hymenea courbaril* Linneaus) occurring in the Brazilian Cerrado: A review. Res. Soc. Dev..

[22] Chavasco J.M., Prado P.B.H.M., Cerdeira C.D., Leandro F.D., Coelho L.F.L., Silva J.J., Chavasco J.K., Dias A.L.T. (2014). Evaluation of Antimicrobial and Cytotoxic Activities of Plant Extracts from the Cerrado of Southern Minas Gerais. Rev. Inst. Med. Trop. Sao Paulo.

[23] Fernandes T.T., Fernandes A.T.S., Pimenta F.C. (2007). Antimicrobial activity of plants *Plathymenia reticulata, Hymenaea courbaril* and *Guazuma ulmifolia*. Rev. Patol. Trop..

[24] Tsuchiya H., Sato M., Miyazaki T., Fujiwara S., Tanigaki S., Ohyama M., Tanaka T., Iinuma M. (1996). Comparative Study on the Antibacterial Activity of Phytochemical Flavanones against Methicillin-Resistant *Staphylococcus aureus*. J. Ethnopharmacol..

[25] Artavia D., Barrios M., Castro O. (1995). Flavanonol Rhamnoside from *Hymenaea courbaril* Leaves. Fitoterapia.

[26] Pettit G.R., Meng Y., Stevenson C.A., Doubek D.L., Knight J.C., Cichacz Z., Pettit R.K., Chapuis J.C., Schmidt J.M. (2003). Isolation and Structure of Palstatin from the Amazon Tree *Hymeneae palustris*. J. Nat. Prod..

[27] Al-Shabib N.A., Husain F.M., Ahmad I., Khan M.S., Khan R.A., Khan J.M. (2017). Rutin Inhibits Mono and Multi-Species Biofilm Formation by Foodborne Drug Resistant *Escherichia coli* and *Staphylococcus aureus*. Food Control.

[28] Di Lodovico S., Bacchetti T., D’Ercole S., Covone S., Petrini M., Di Giulio M., Di Fermo P., Diban F., Ferretti G., Cellini L. (2022). Complex Chronic Wound Biofilms Are Inhibited in Vitro by the Natural Extract of *Capparis spinose*. Front. Microbiol..

[29] Kang S.S., Kim J.-G., Lee T.-H., Oh K.-B. (2006). Flavonols Inhibit Sortases and Sortase-Mediated *Staphylococcus aureus* Clumping to Fibrinogen. Biol. Pharm. Bull..

[30] Cho H.S., Lee J.H., Cho M.H., Lee J. (2015). Red Wines and Flavonoids Diminish *Staphylococcus aureus* Virulence with Anti-Biofilm and Anti-Hemolytic Activities. Biofouling.

[31] Cowan M.M. (1999). Plant Products as Antimicrobial Agents. Clin. Microbiol. Rev..

[32] Wijesundara N.M., Vasantha Rupasinghe H.P. (2019). Bactericidal and Anti-Biofilm Activity of Ethanol Extracts Derived from Selected Medicinal Plants against *Streptococcus pyogenes*. Molecules.

[33] Sá M.C.A., Peixoto R.M., Krewer C.C., Almeida J.R.G.S., Vargas A.C., Costa M.M. (2011). Antimicrobial Activity of Caatinga Biome Ethanolic Plant Extracts against Gram Negative and Positive Bacteria. Rev. Bras. Ciênc. Vet..

[34] Singleton V.L., Rossi J.A. (1965). Colorimetry of Total Phenolics with Phosphomolybdic-Phosphotungstic Acid Reagents. Am. J. Enol. Vitic..

[35] Prieto P., Pineda M., Aguilar M. (1999). Spectrophotometric Quantitation of Antioxidant Capacity through the Formation of a Phosphomolybdenum Complex: Specific Application to the Determination of Vitamin E. Anal. Biochem..

[36] Oyaizu M. (1986). Studies on Products of Browning Reaction. Antioxidative Activities of Products of Browning Reaction Prepared from Glucosamine. Jpn. J. Nutr. Diet..

[37] (2018). Performance Standards for Antimicrobial Disk Susceptibility Testing; Approved Standard.

[38] Ferreira L.M.R., Azevedo X.E.C.P., Luna J.S., Goulart S.A.E. (2006). The Antibiotic Activity of Some Brazilian Medicinal Plants. Braz. J. Pharmacog..

[39] Brambilla L.Z.S., Endo E.H., Cortez D.A.G., Filho B.P.D. (2017). Anti-Biofilm Activity against *Staphylococcus aureus* MRSA and MSSA of Neolignans and Extract of *Piper regnellii*. Braz. J. Pharmacog..

