# Supplementary material for: Evaluation of the Antioxidant, Antimicrobial, and Anti-Biofilm Effects of the Stem Bark, Leaf, and Seed Extracts from Hymenaea courbaril and Characterization by UPLC-ESI-QTOF-MS/MS Analysis
Source: Antibiotics (Basel). 2023 Nov 8;12(11):1601. doi: 10.3390/antibiotics12111601 (PMC10668761; doi:10.3390/antibiotics12111601)

SMJL (negative ionization mode)

Fig. S1. MS^2 spectrum of 341.0149 *m/z* [M-H]$^-$ in SMJL extract.

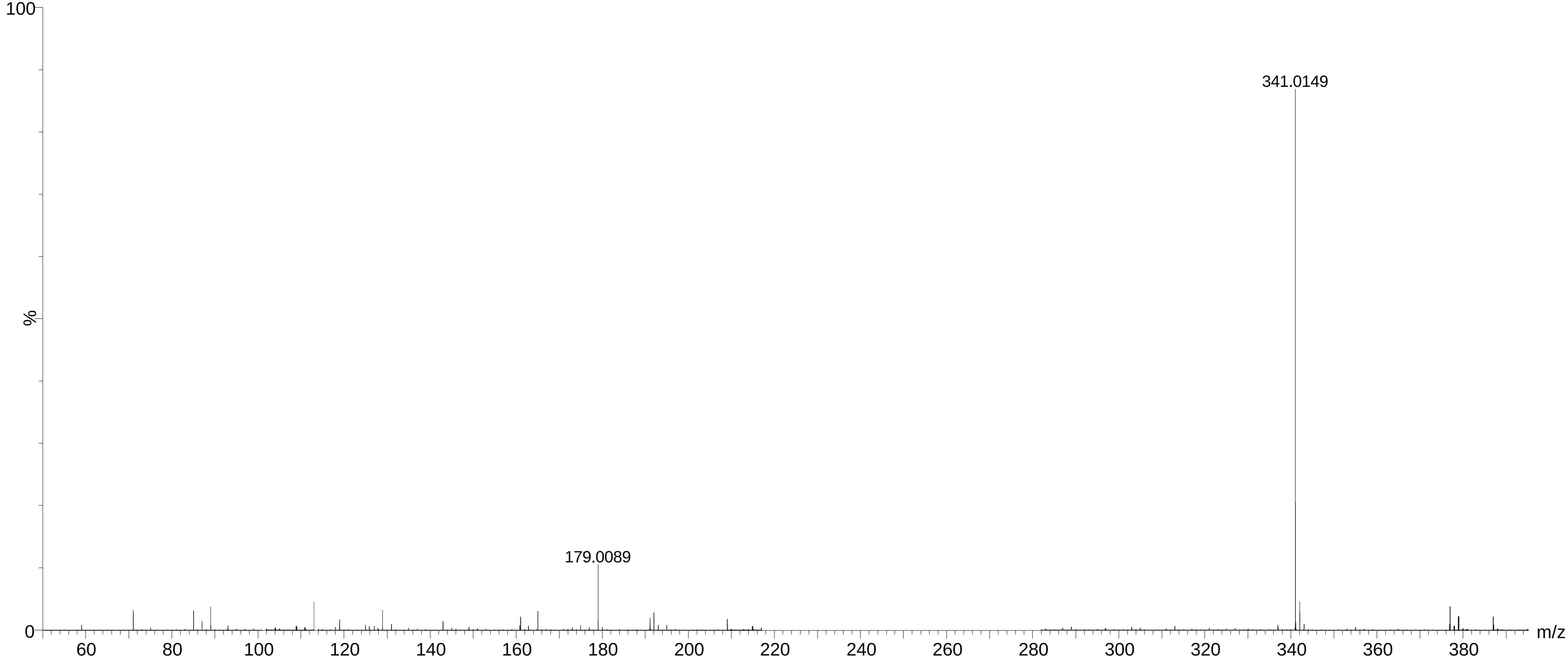

Fig. S2. MS^2 spectrum of 179.0089 *m/z* [M-H]$^-$ in SMJL extract.

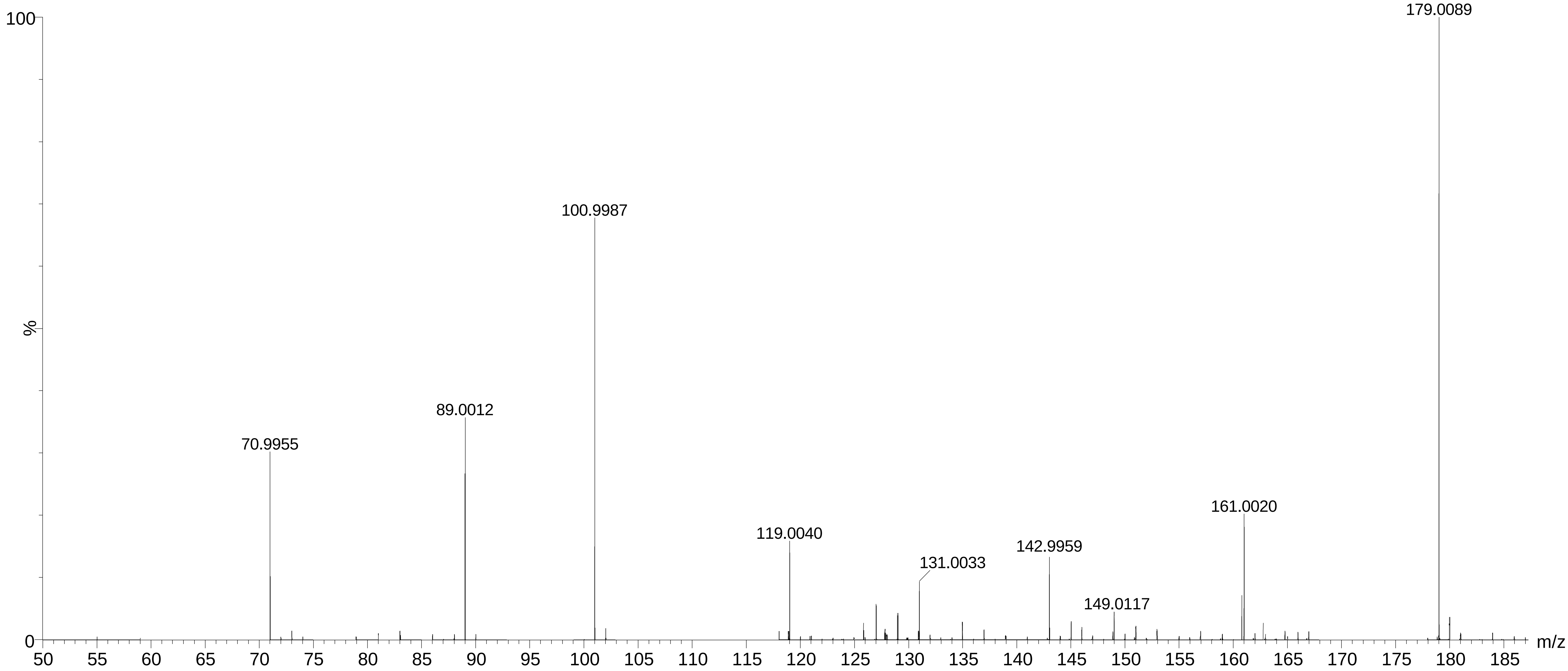

Fig. S3. MS^2 spectrum of 607.9508 *m/z* [M-H]$^-$ in SMJL extract.

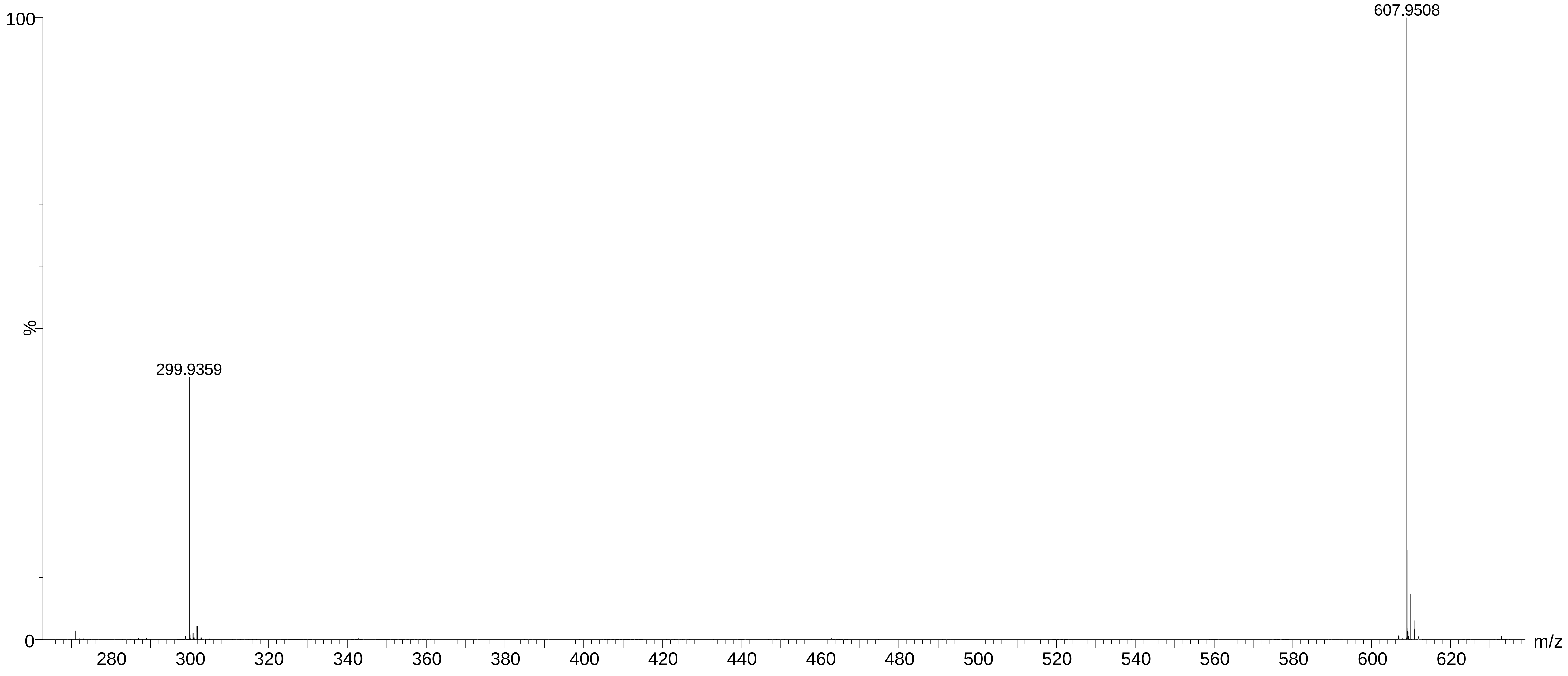

Fig. S4. MS^2 spectrum of 609.9561 *m/z* [M-H]$^-$ in SMJL extract.

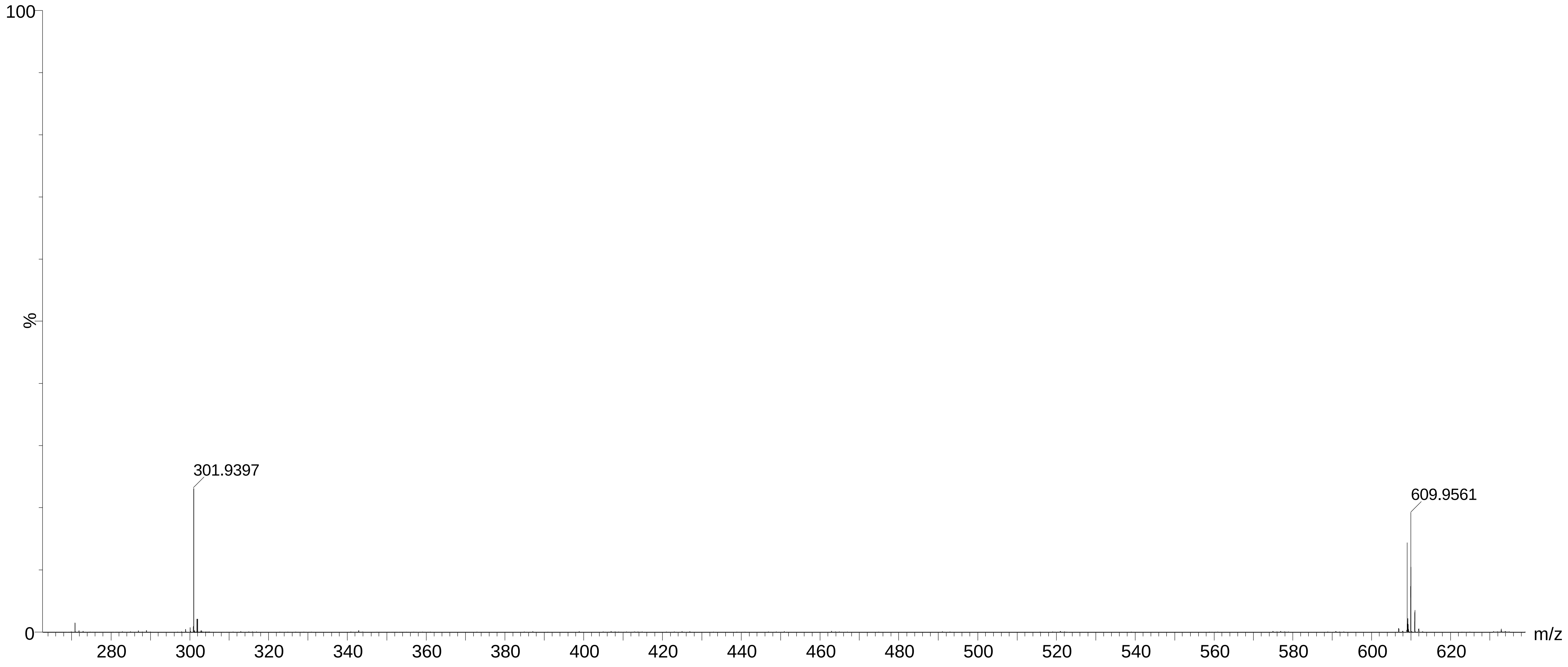

Fig. S5. MS^2 spectrum of 593.0129 *m/z* [M-H]$^-$ in SMJL extract.

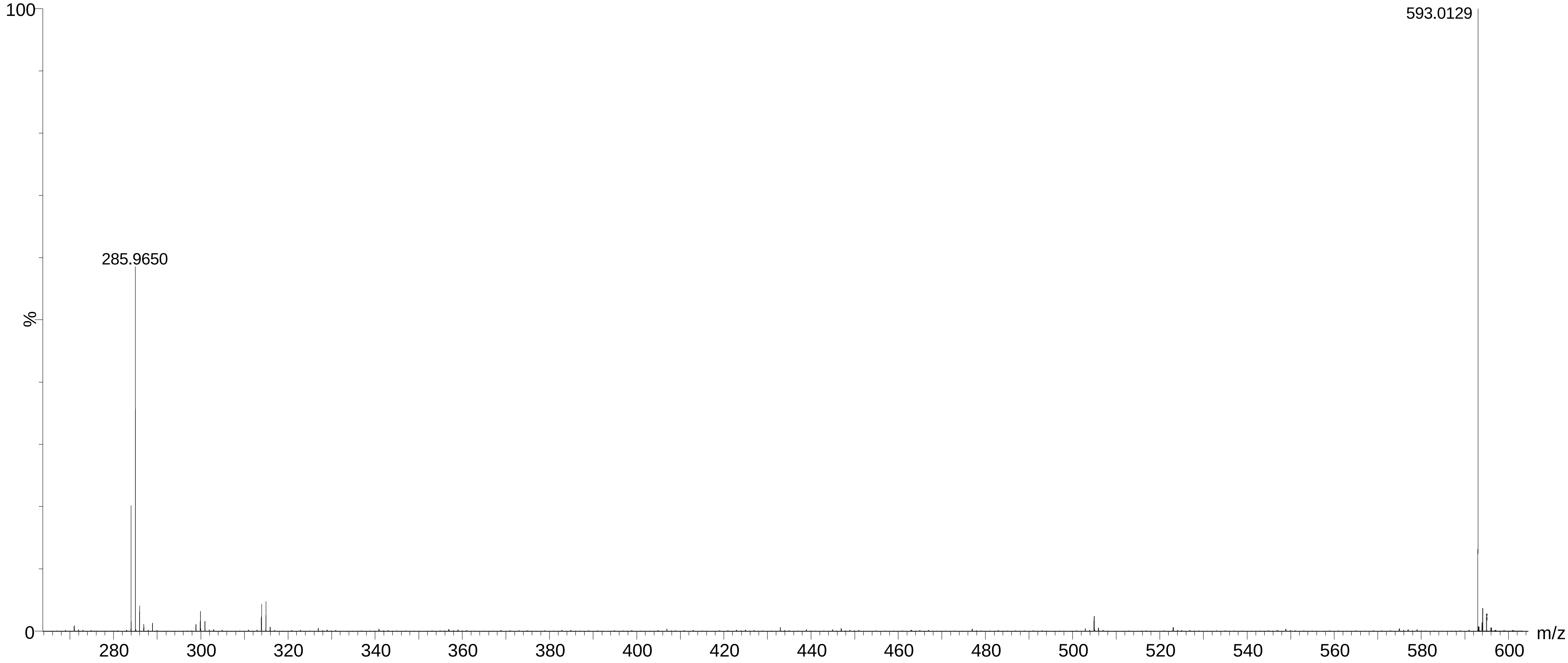

Fig. S6. MS^2 spectrum of 623.0092 *m/z* [M-H]$^-$ in SMJL extract.

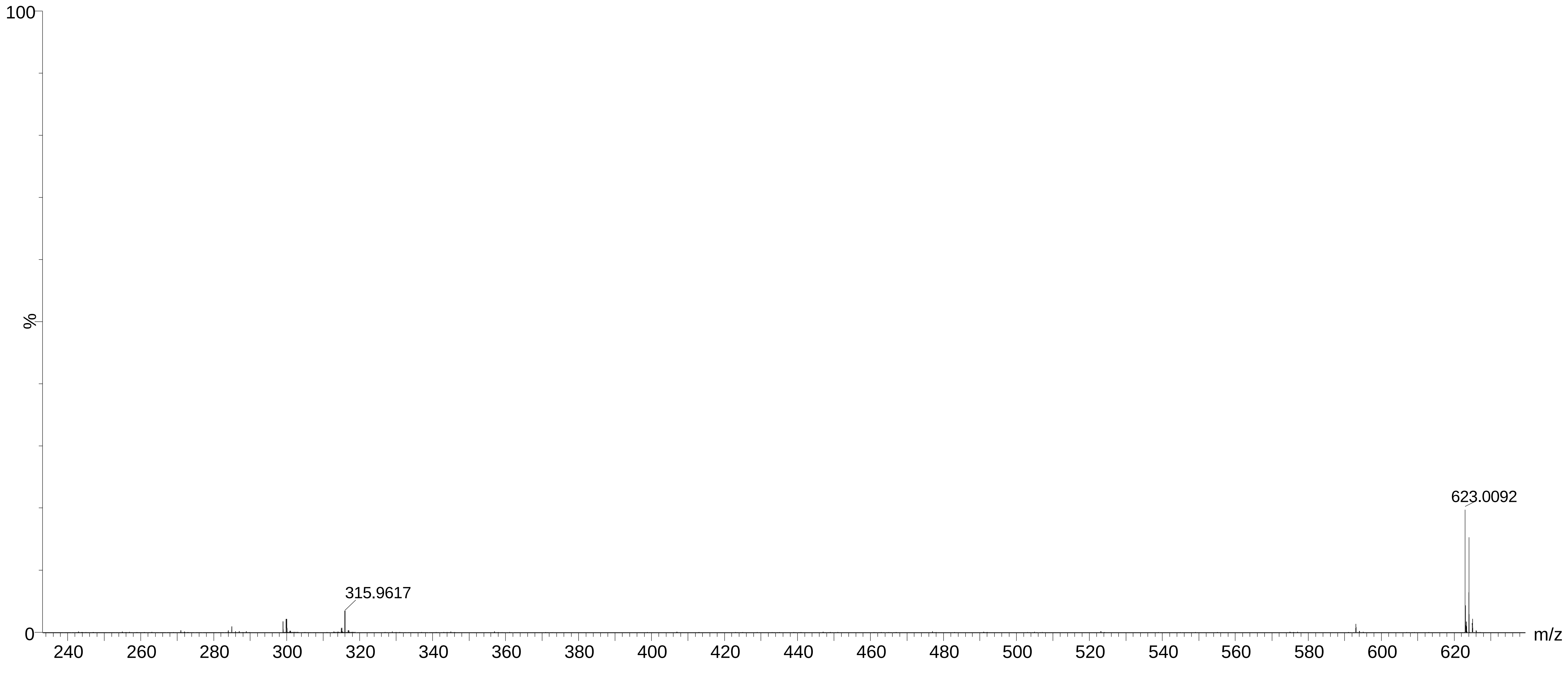

Fig. S7. MS^2 spectrum of 477.9869 *m/z* [M-H]$^-$ in SMJL extract.

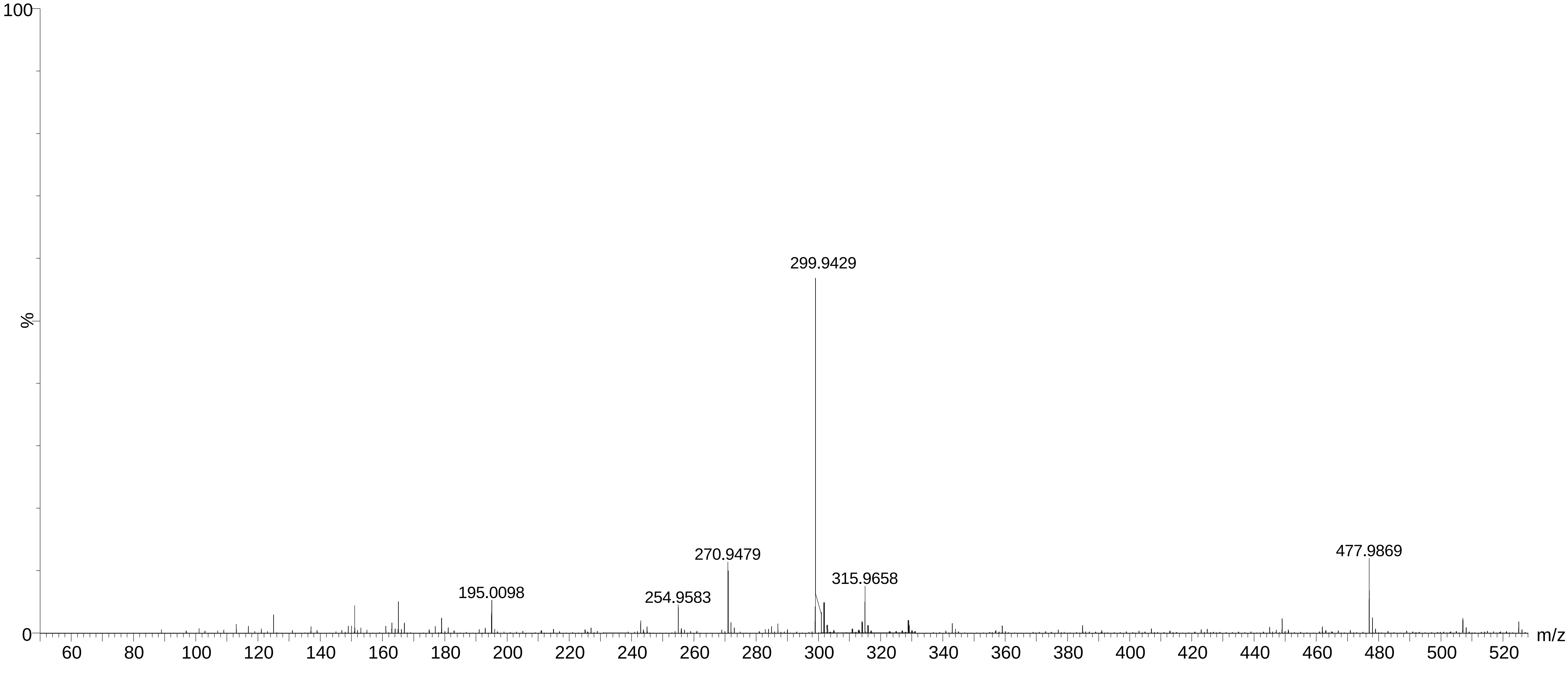

Fig. S8. MS^2 spectrum of 329.9728 *m/z* [M-H]$^-$ in SMJL extract.

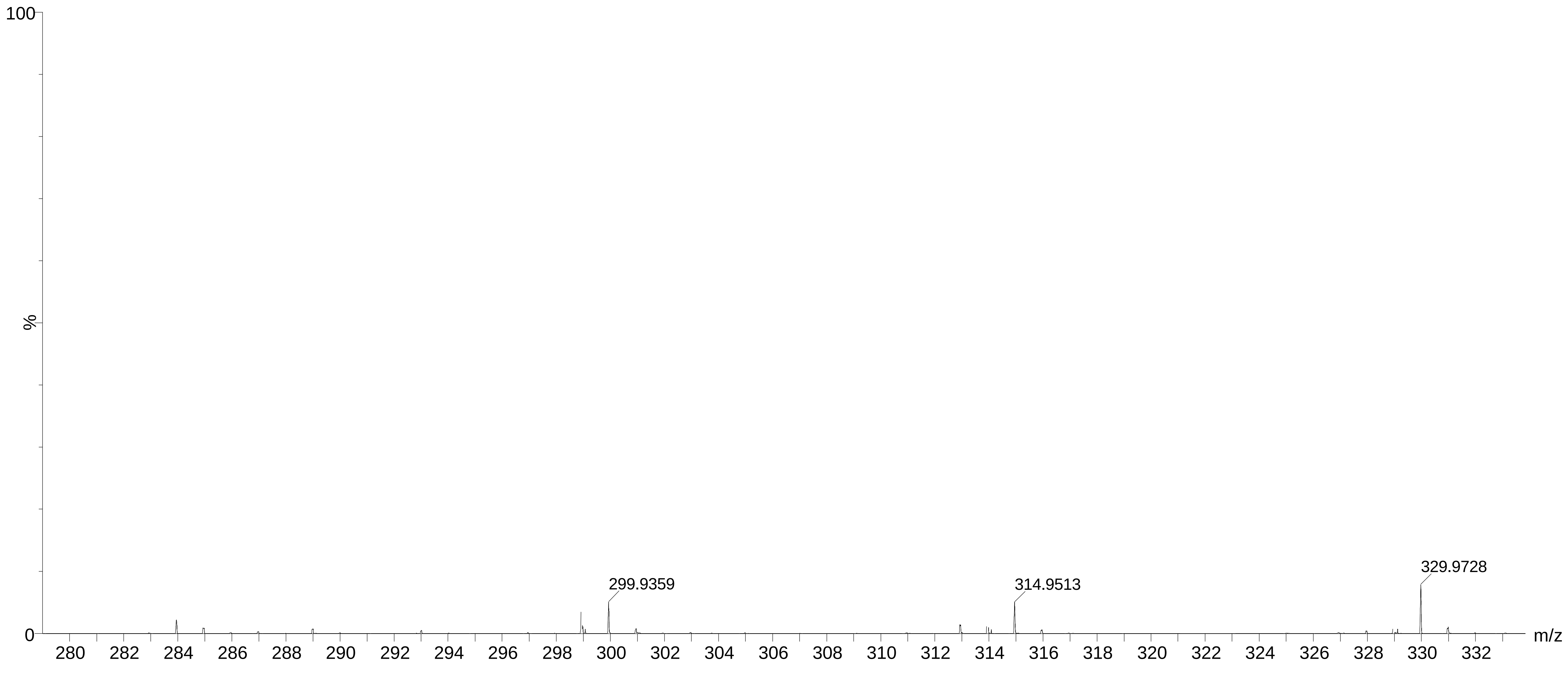

Fig. S9. MS^2 spectrum of 313.9497 *m/z* [M-H]$^-$ in SMJL extract.

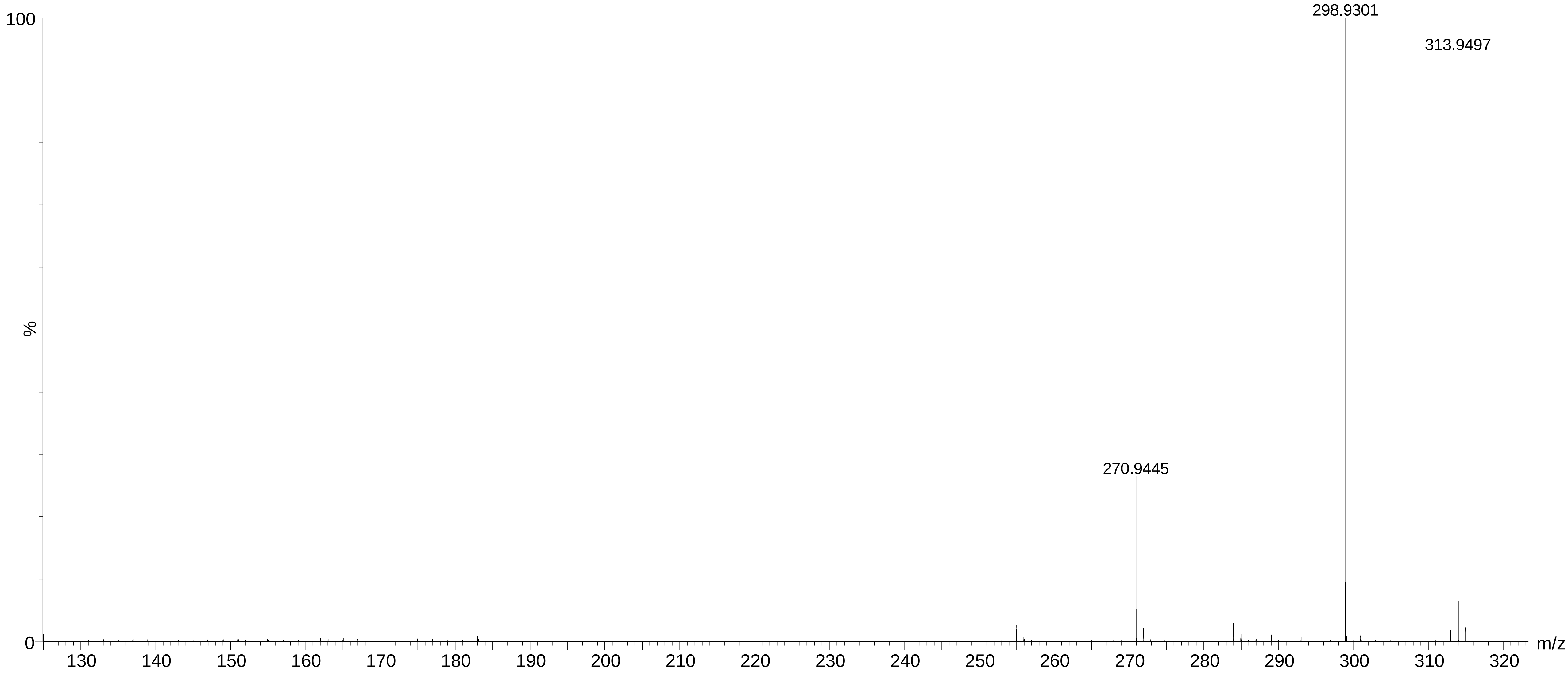

Fig. S10. MS^2 spectrum of 317.1195 *m/z* [M-H]$^-$ in SMJL extract.

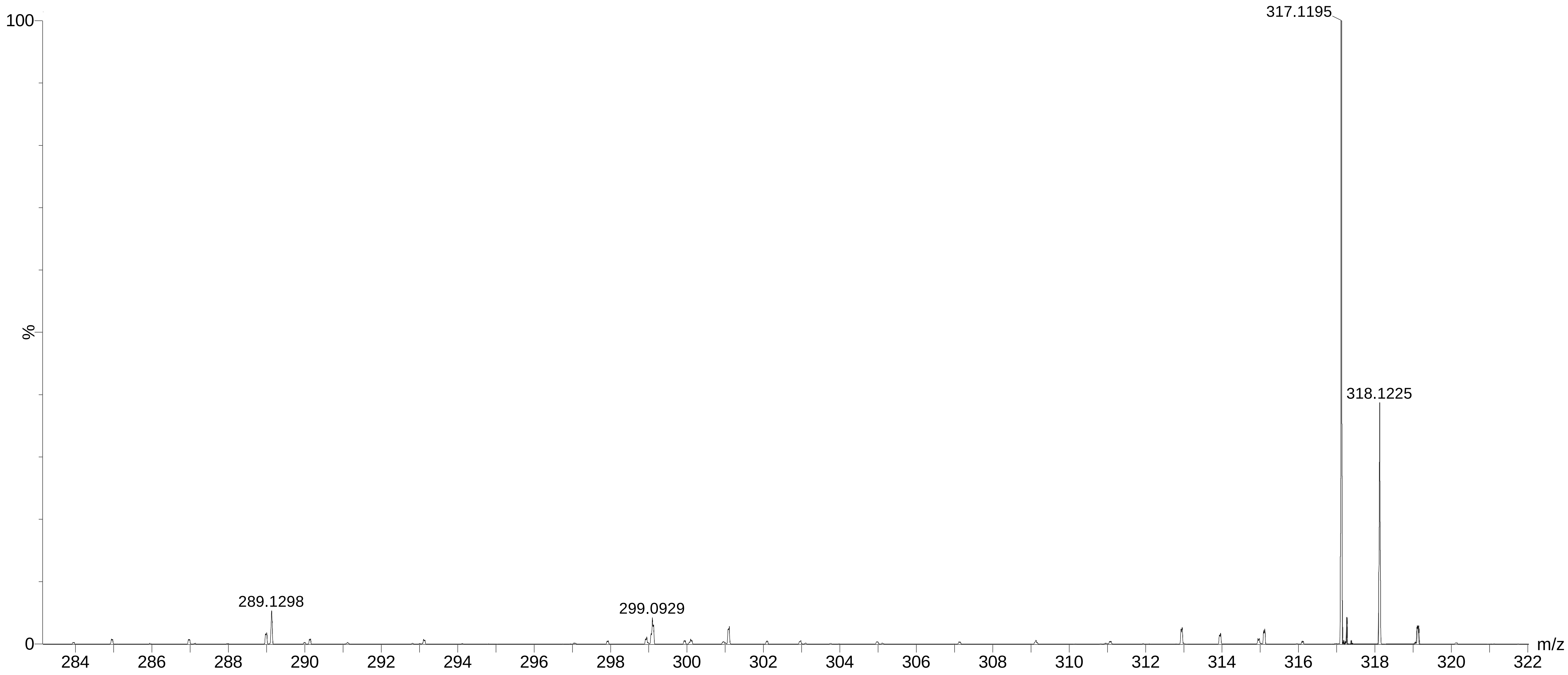

Fig. S11. MS^2 spectrum of 633.3182 *m/z* [M-H]$^-$ in SMJL extract.

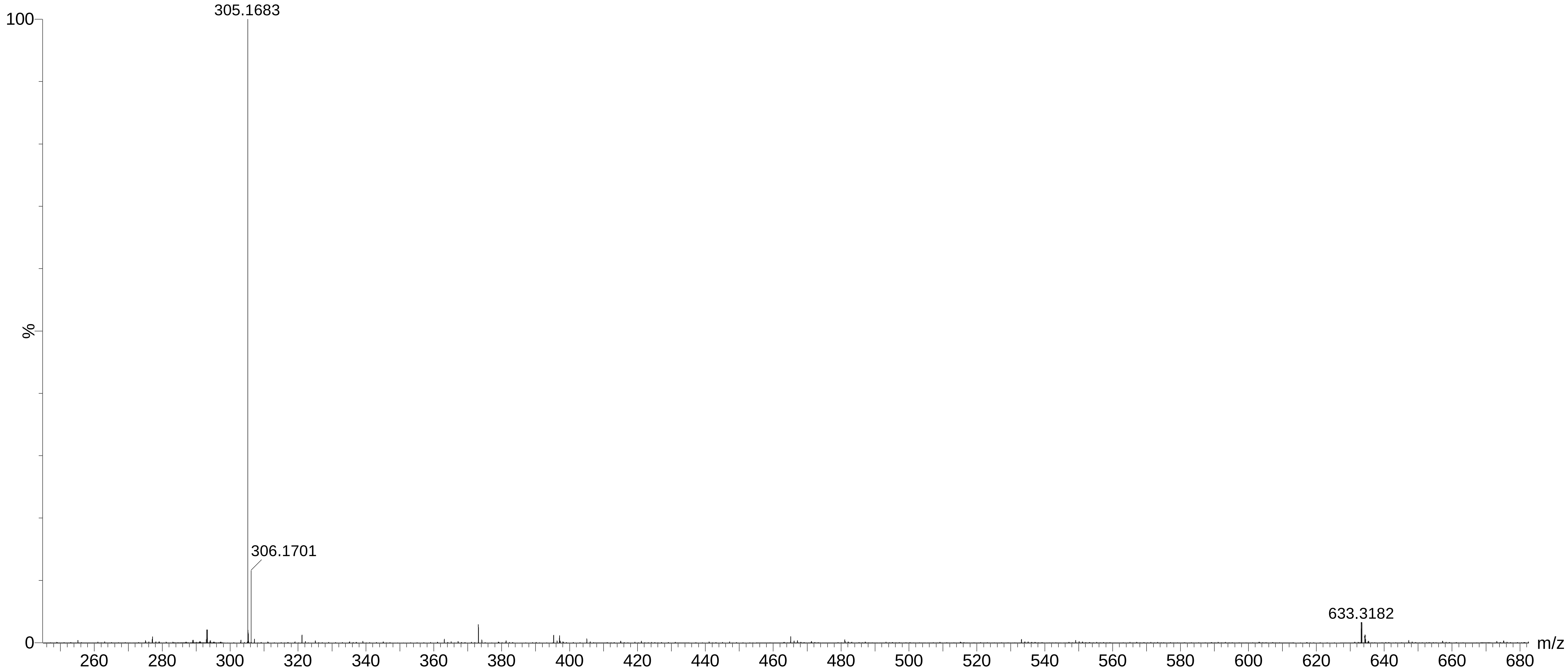

SMJL (positive ionization mode)

Fig. S12. MS² spectrum of 633.7400 *m/z* [M+Na]⁺ in SMJL extract.

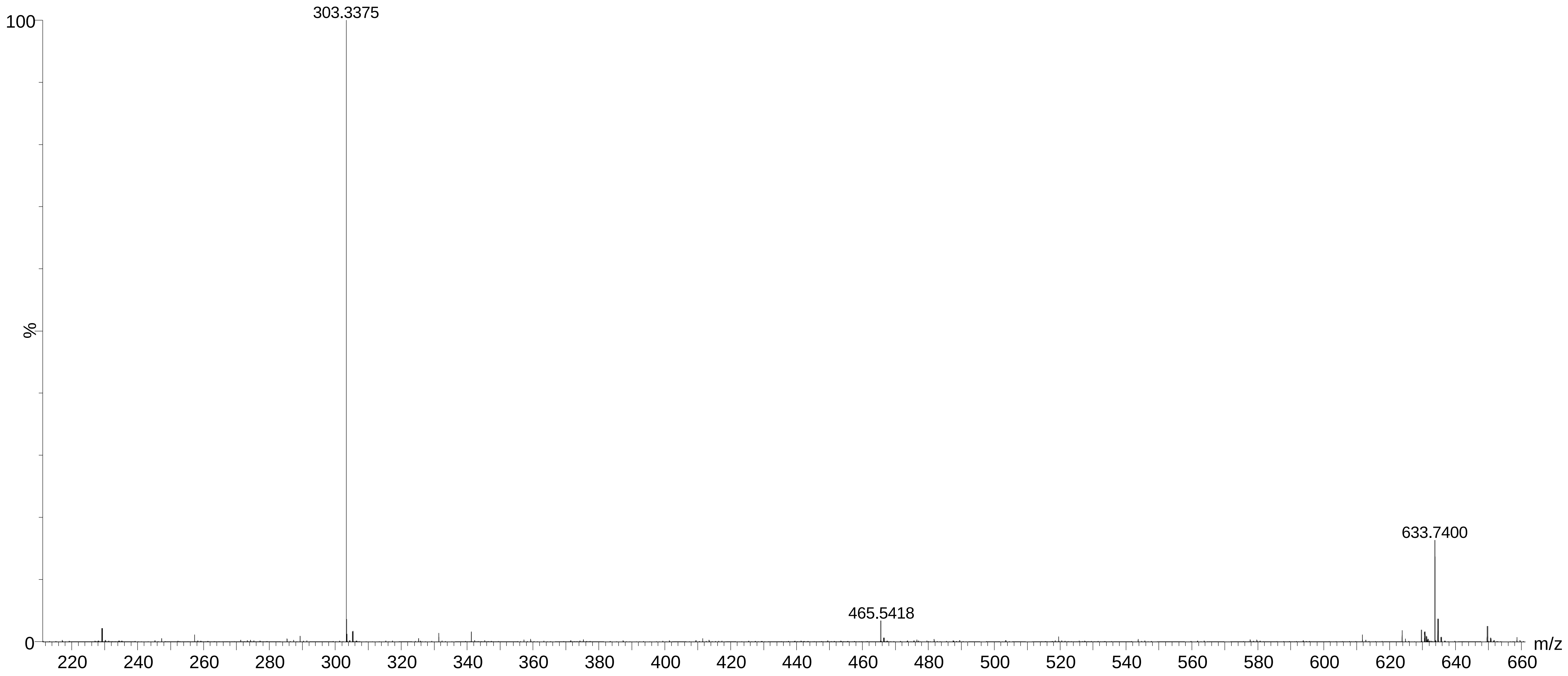

Fig. S13. MS^2 spectrum of 487.5446 *m/z* [M+Na]$^+$ in SMJL extract.

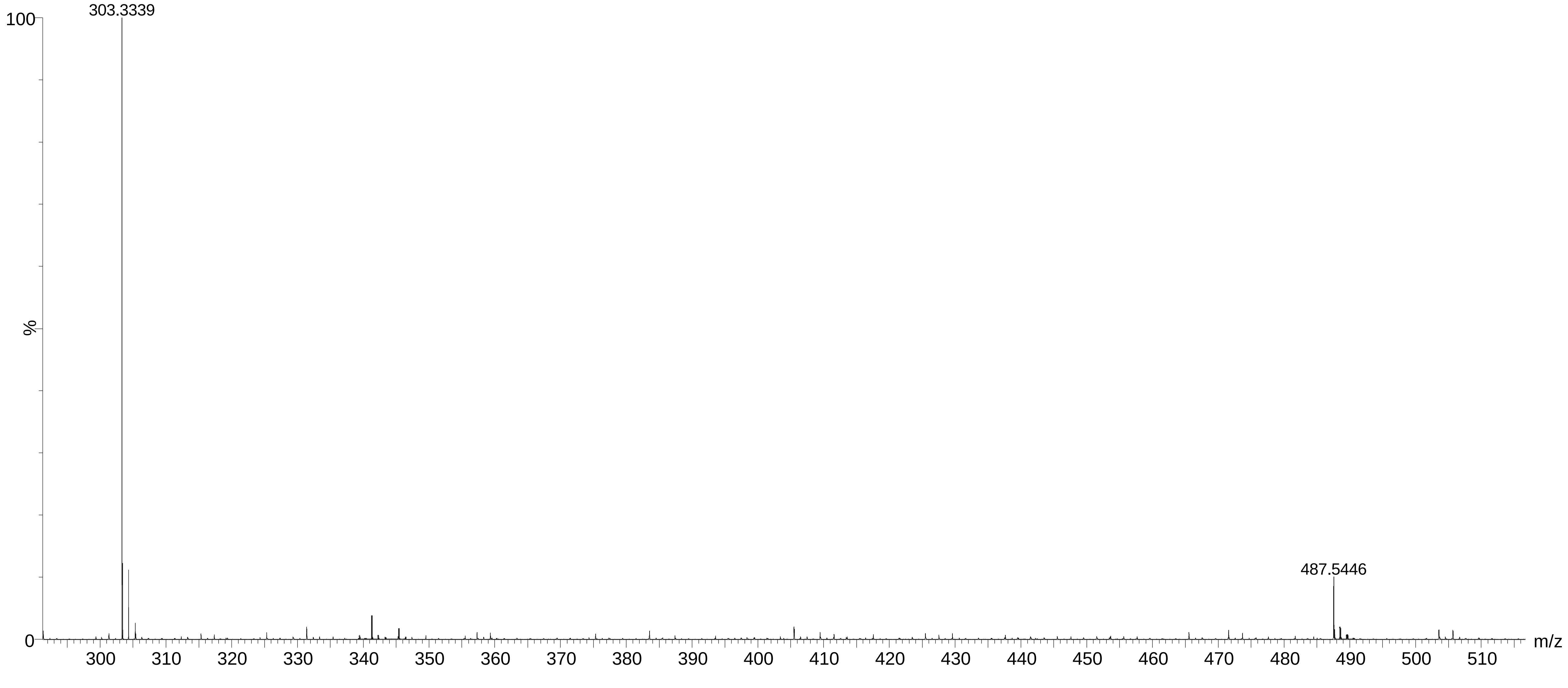

Fig. S14. MS^2 spectrum of 317.3806 *m/z* [M+H]$^+$ in SMJL extract.

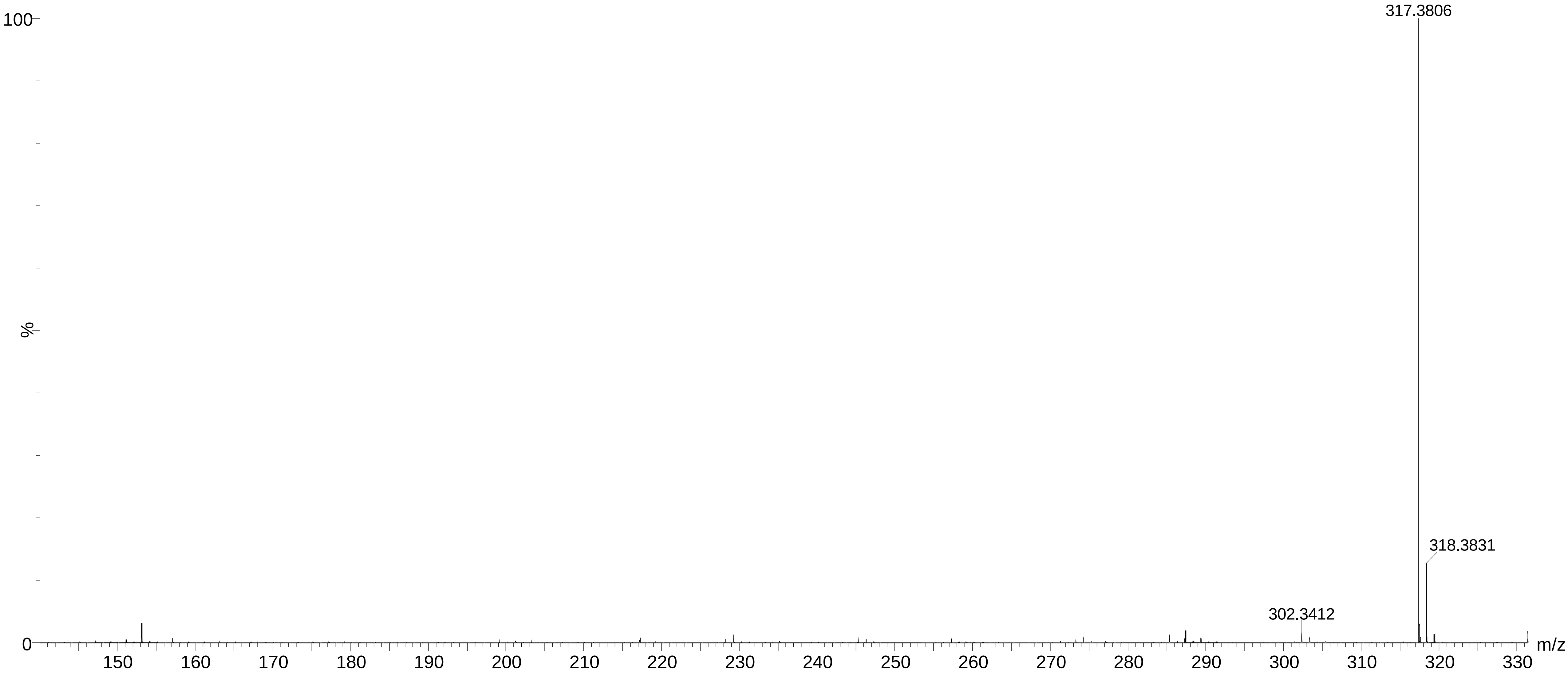

UBJL (negative ionization mode)

Fig. S15. MS^2 spectrum of 341.0376 *m/z* [M-H]$^-$ in UBJL extract.

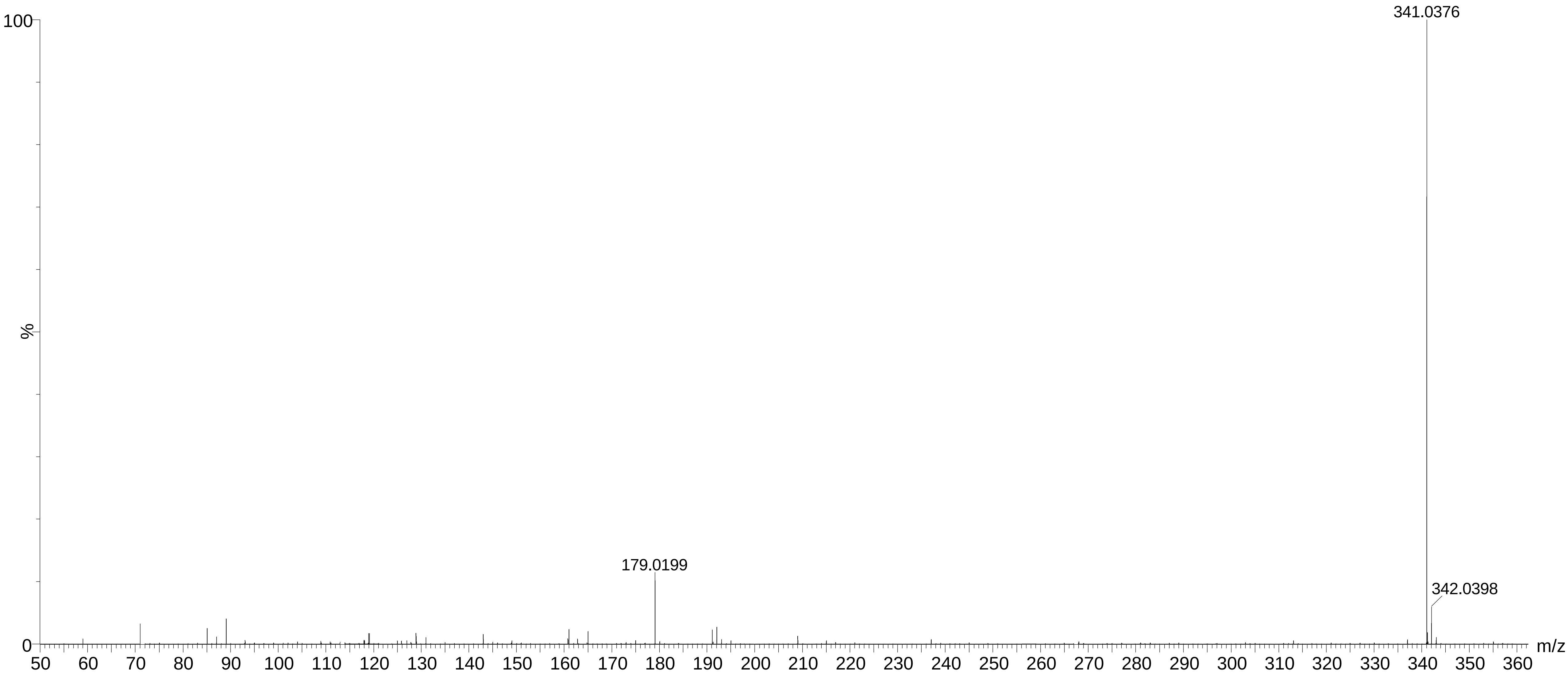

Fig. S16. MS^2 spectrum of 179.0199 *m/z* [M-H]$^-$ in UBJL extract.

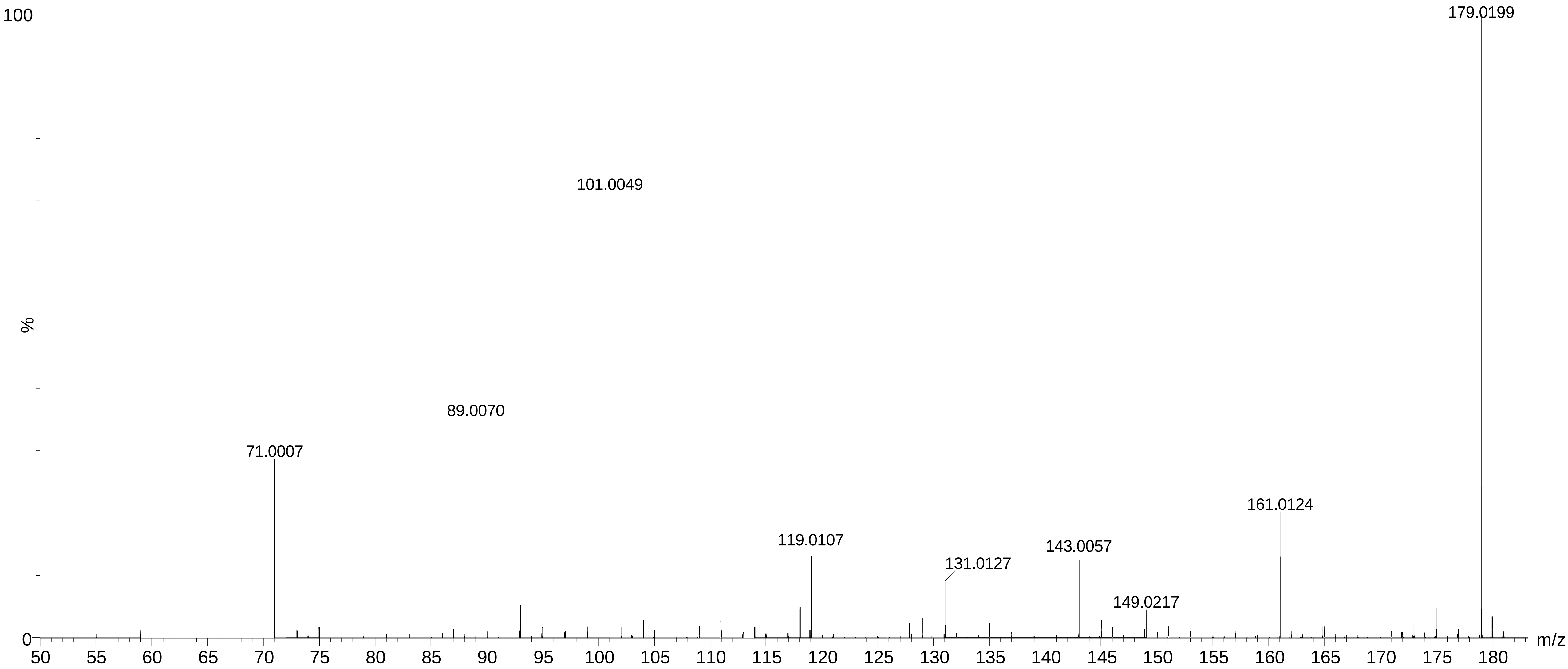

Fig. S17. MS^2 spectrum of 607.9912 *m/z* [M-H]$^-$ in UBJL extract.

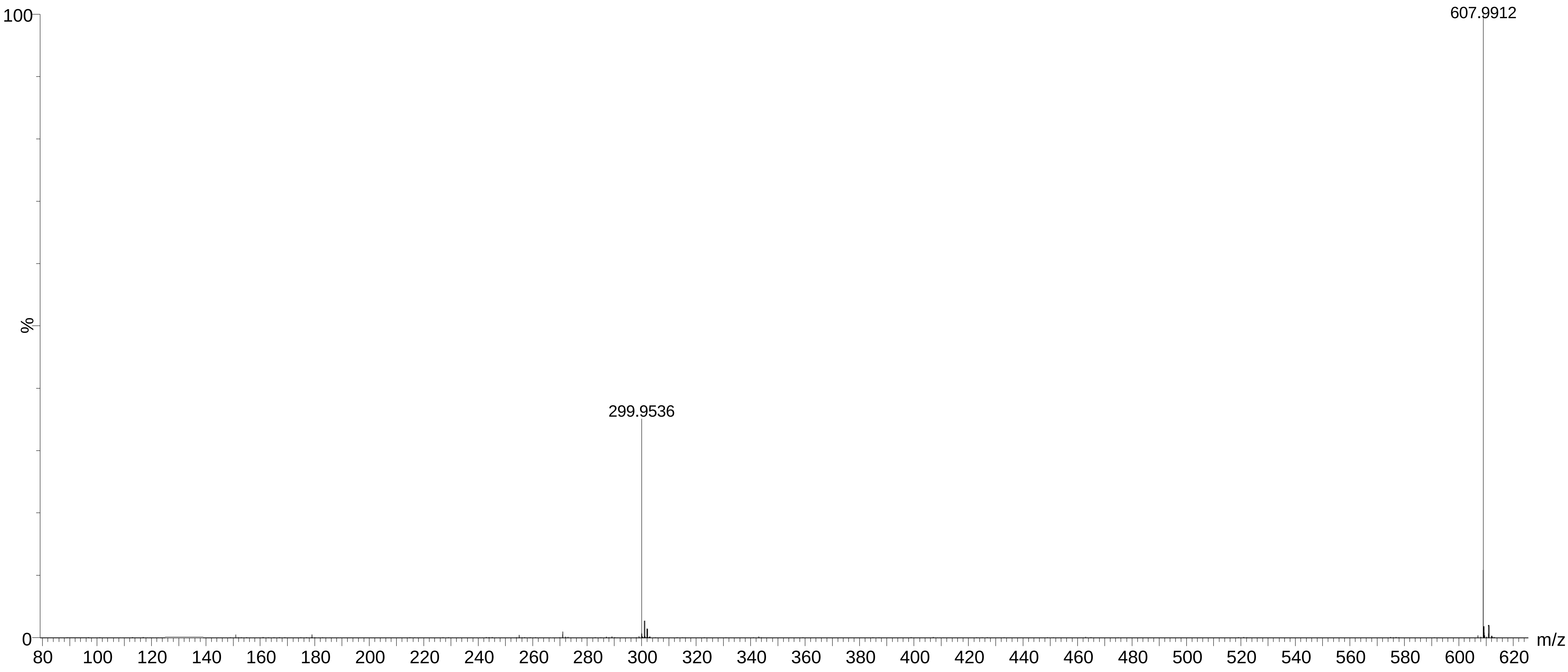

Fig. S18. MS^2 spectrum of 609.9966 *m/z* [M-H]$^-$ in UBJL extract.

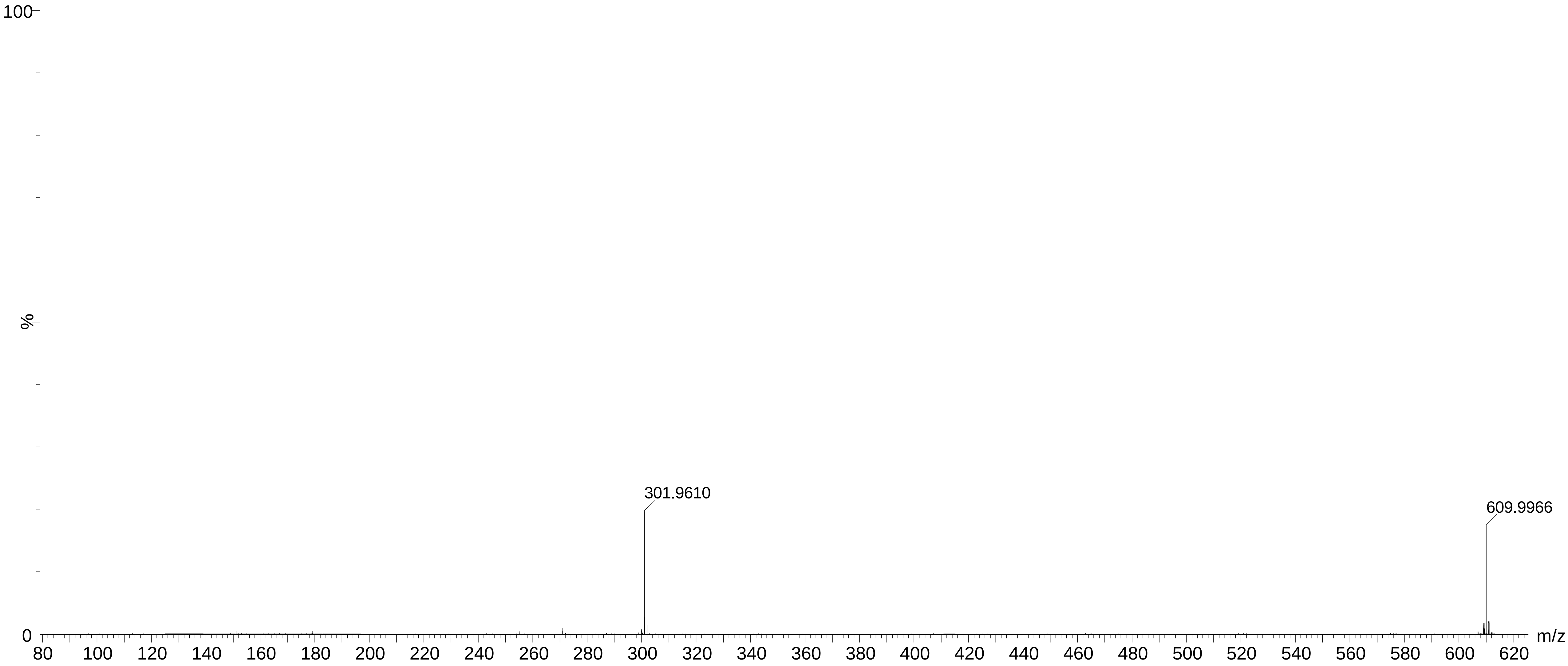

Fig. S19. MS^2 spectrum of 623.0296 *m/z* [M-H]$^-$ in UBJL extract.

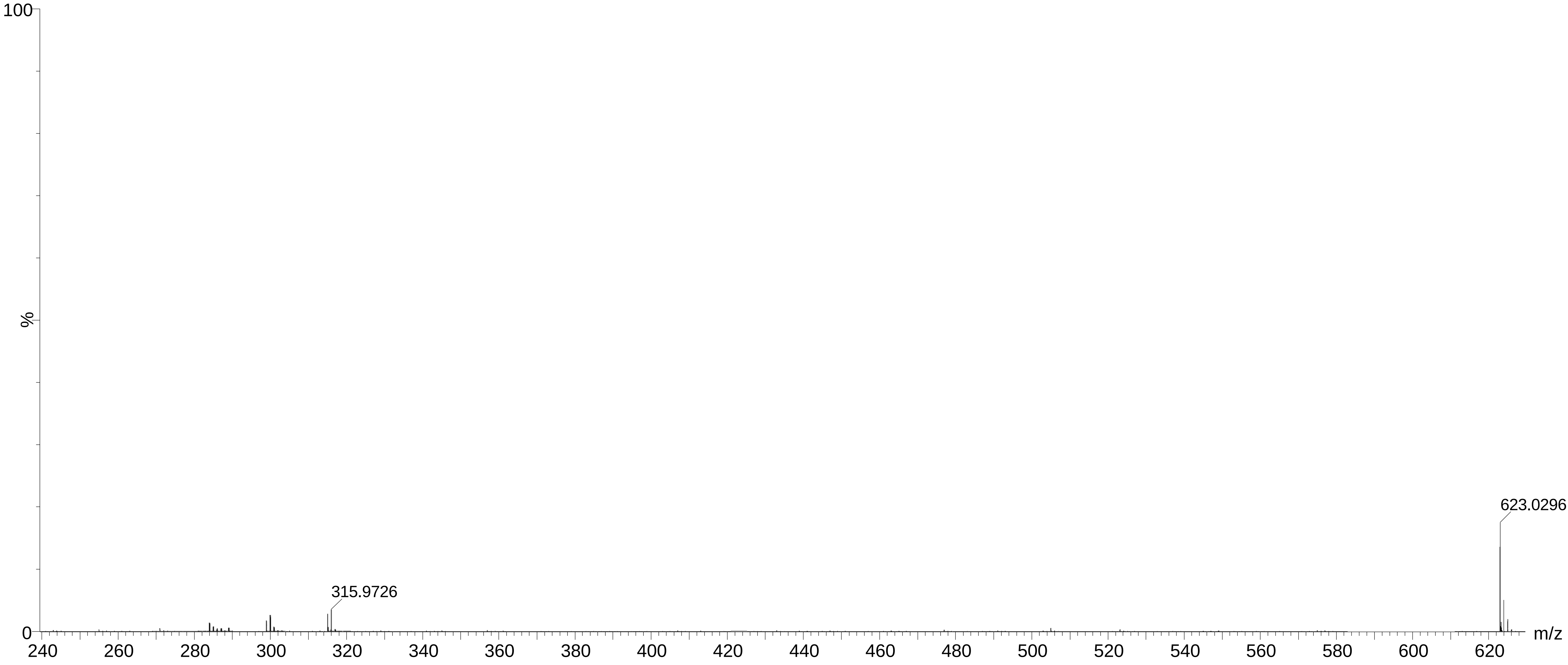

Fig. S20. MS^2 spectrum of 285.9754 *m/z* [M-H]$^-$ in UBJL extract.

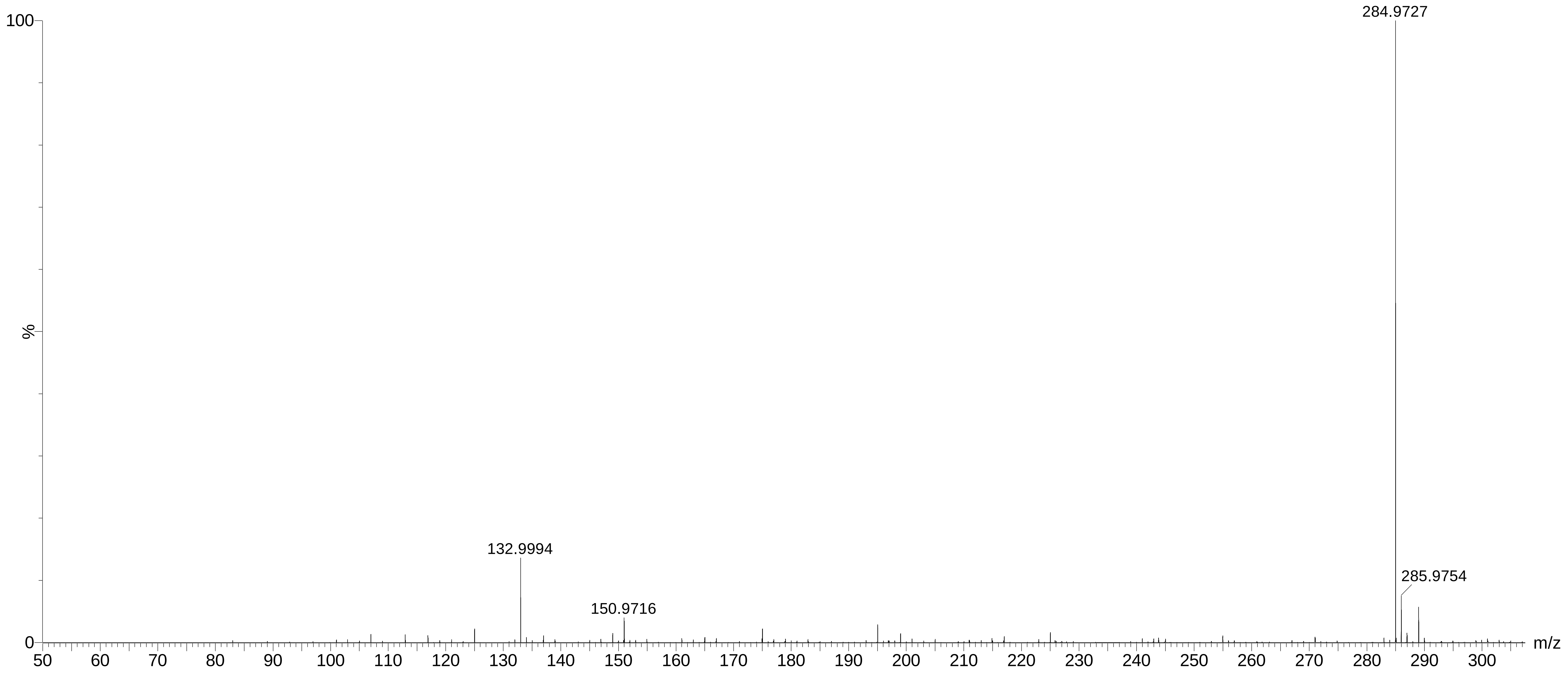

Fig. S21. MS^2 spectrum of 313.9642 *m/z* [M-H]$^-$ in UBJL extract.

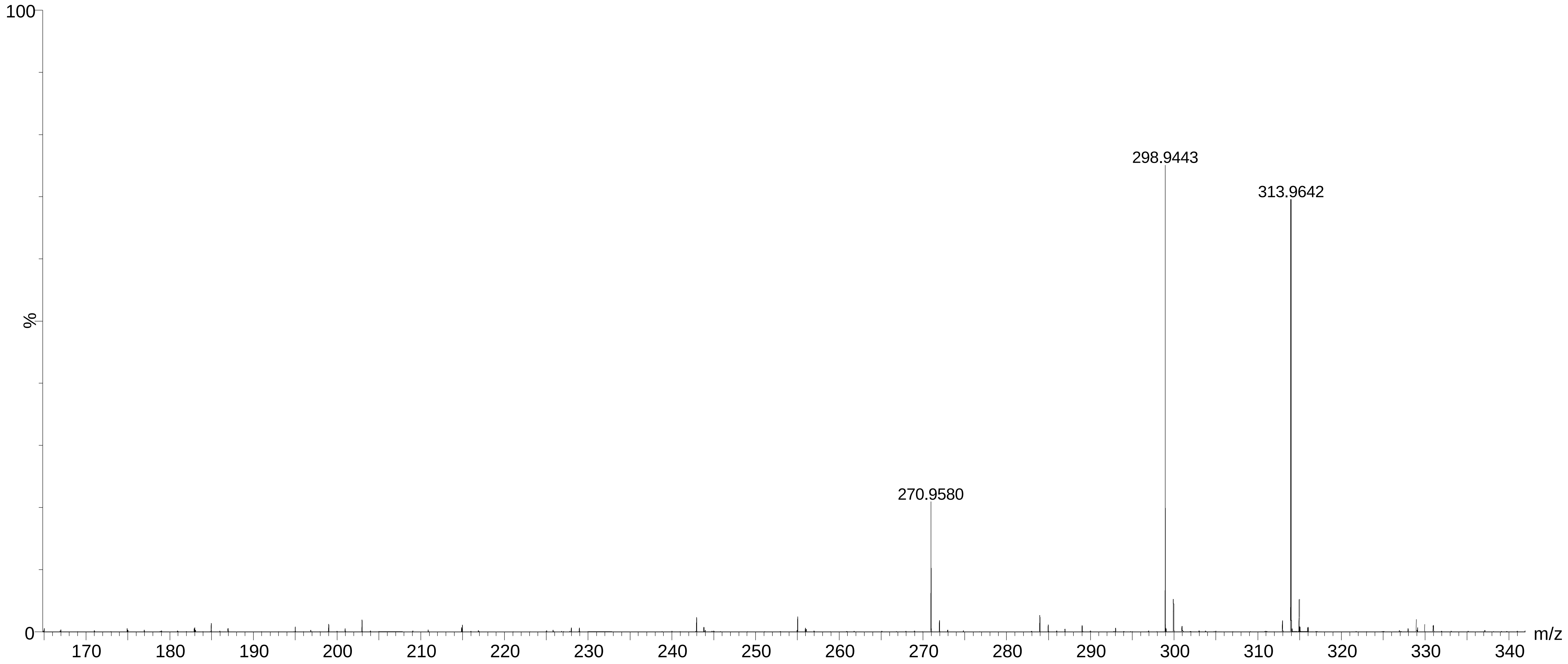

Fig. S22. MS^2 spectrum of 329.9876 *m/z* [M-H]$^-$ in UBJL extract.

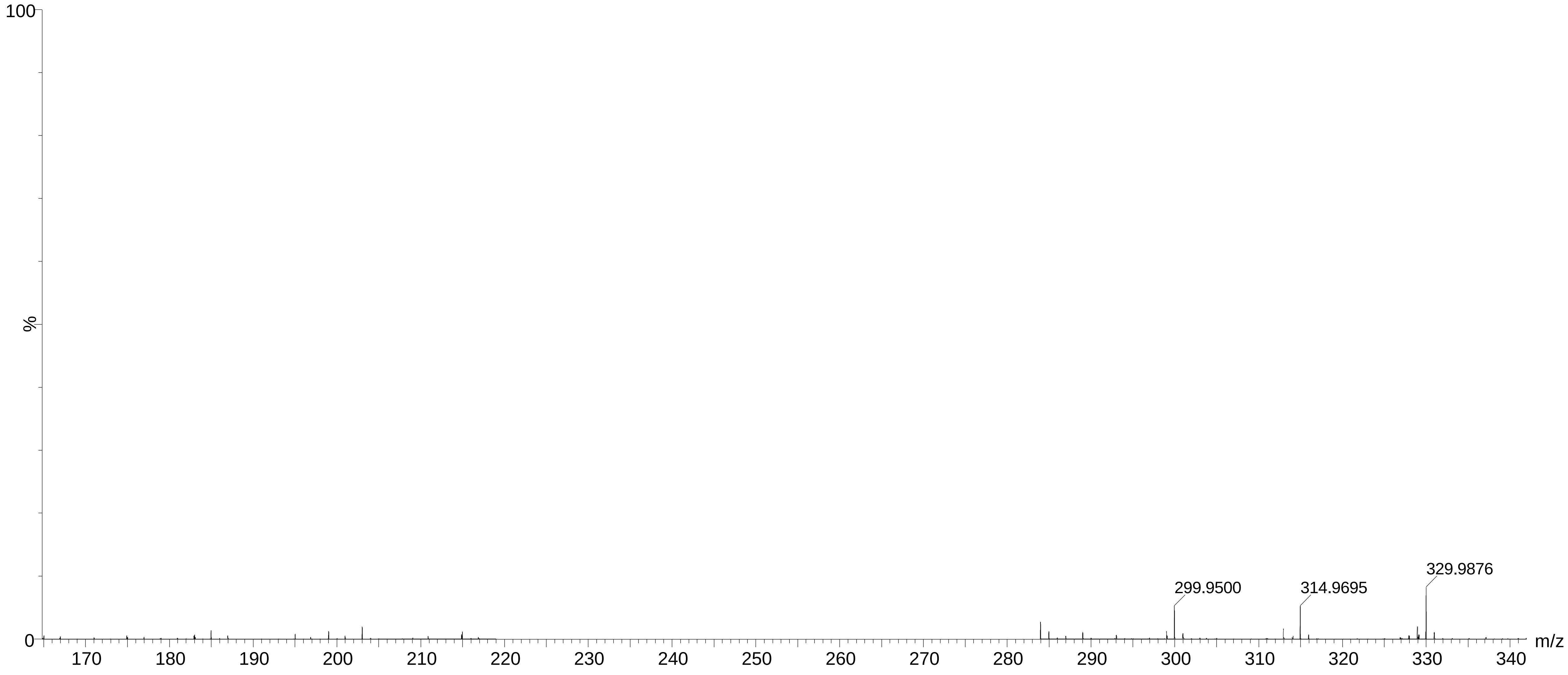

UBJL (positive ionization mode)

Fig. S23. MS^2 spectrum of 633.1422 *m/z* [M+Na]$^+$ in UBJL extract.

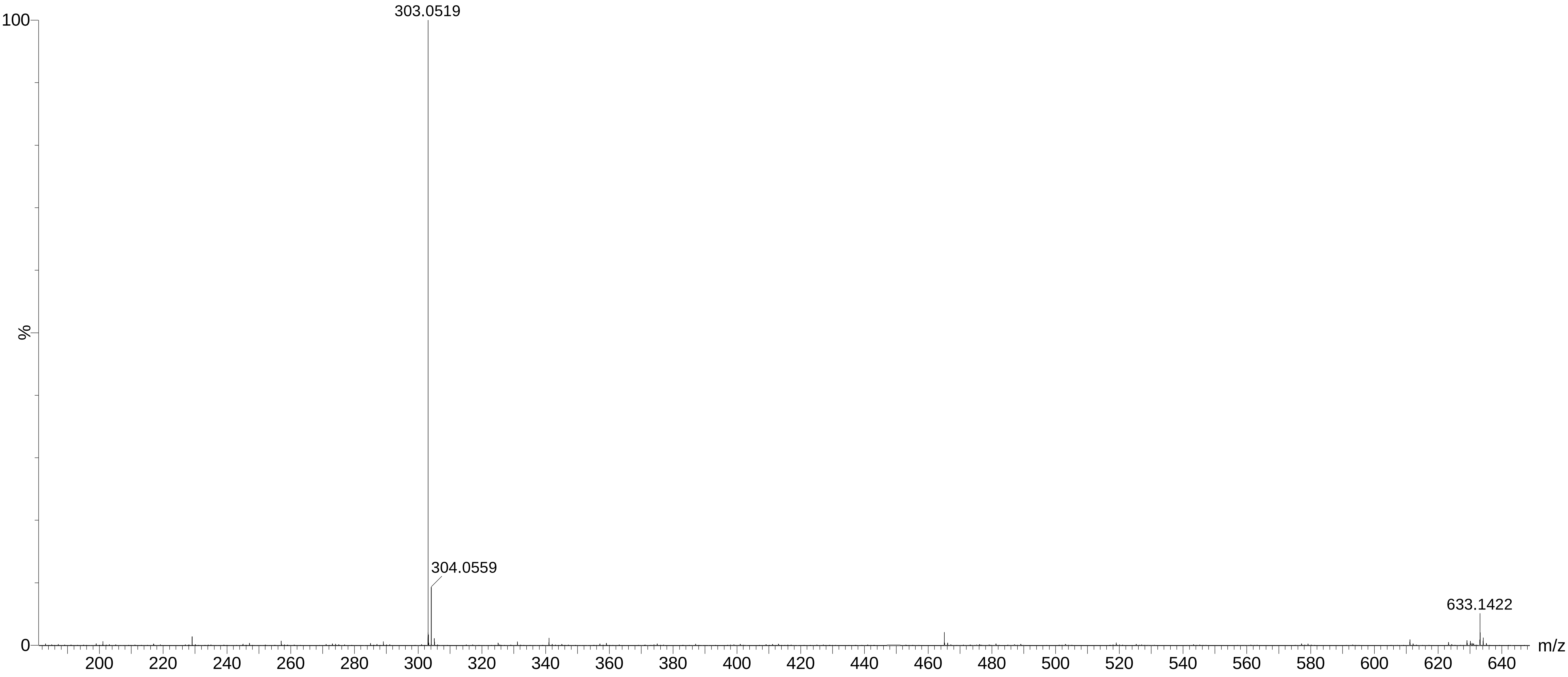

Fig. S24. MS^2 spectrum of 487.0886 *m/z* [M+Na]$^+$ in UBJL extract.

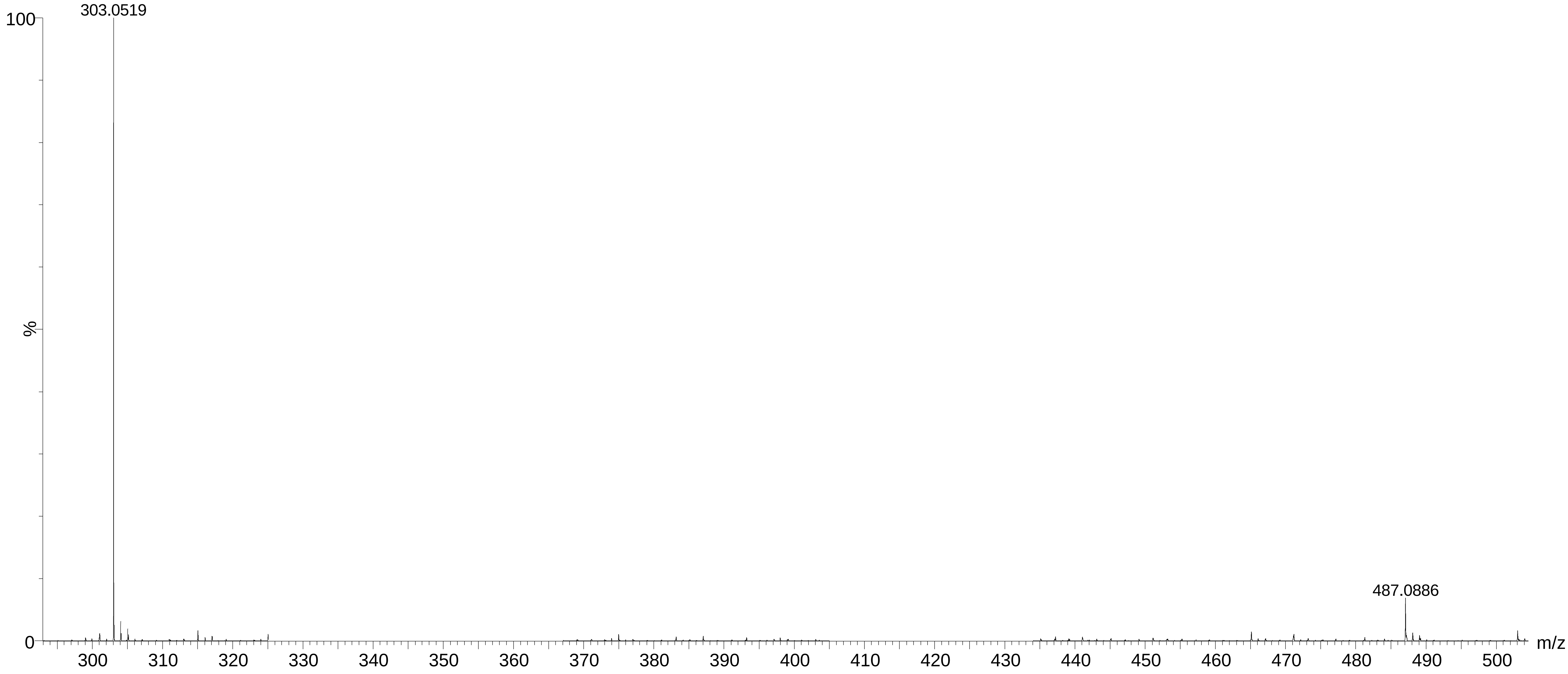

Fig. S25. MS^2 spectrum of 317.0632 *m/z* [M+H]$^+$ in UBJL extract.

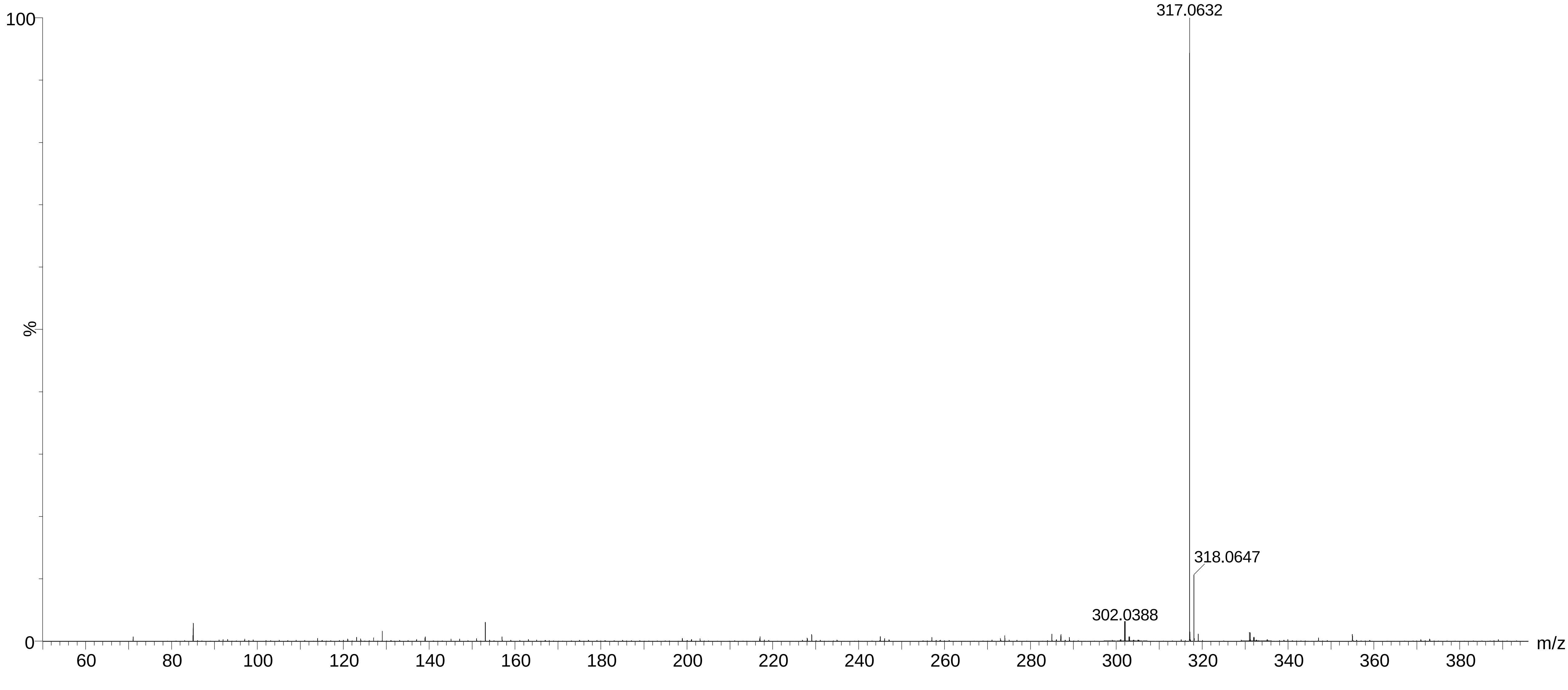

MSJL (negative ionization mode)

Fig. S26. MS^2 spectrum of 341.0451 *m/z* [M-H]$^-$ in MSJL extract.

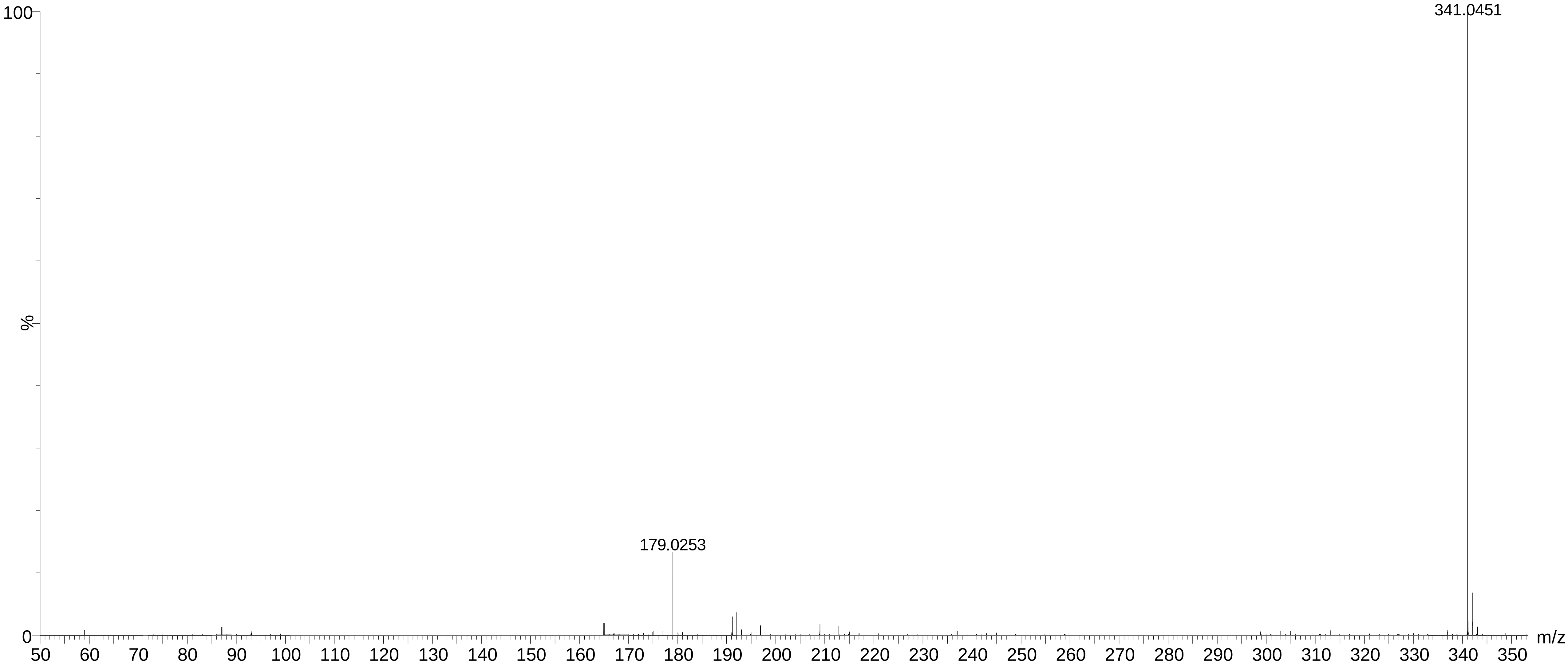

Fig. S27. MS^2 spectrum of 179.0253 *m/z* [M-H]$^-$ in MSJL extract.

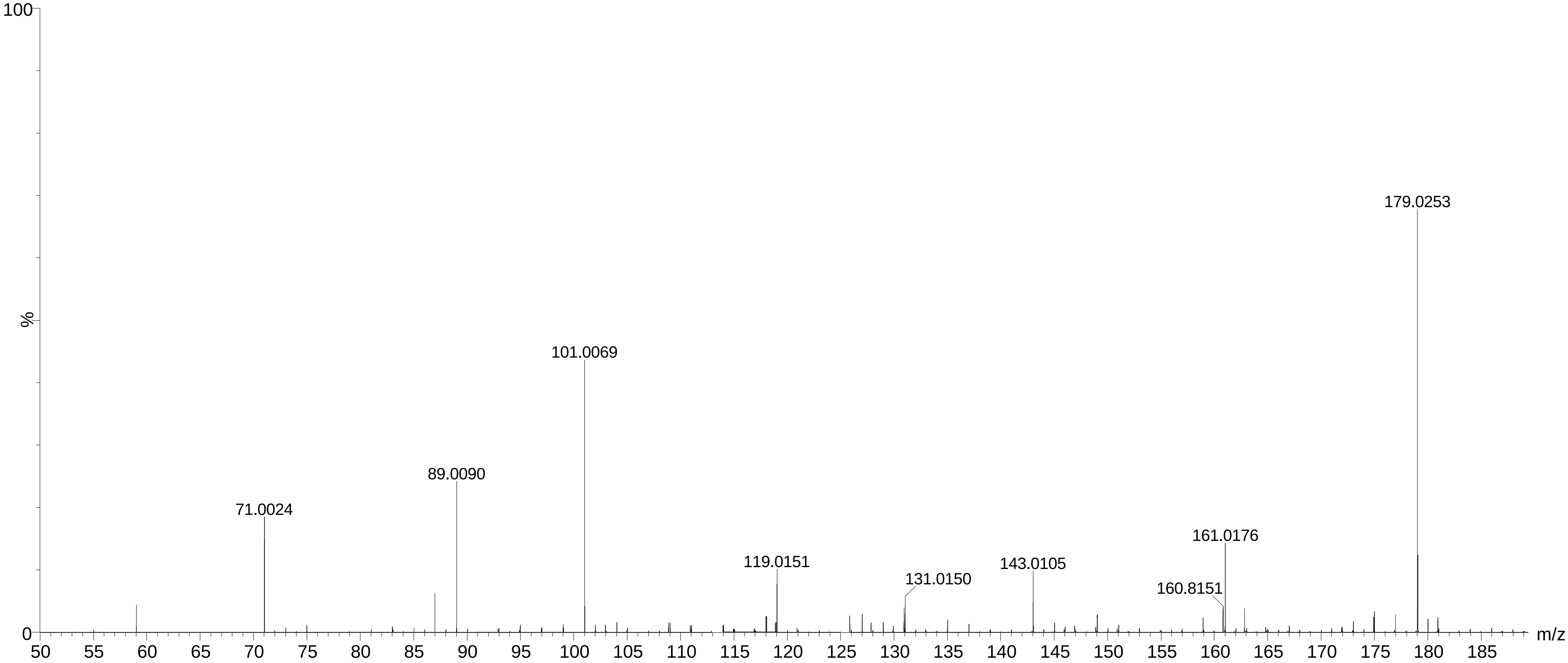

Fig. S28. MS^2 spectrum of 609.0568 *m/z* [M-H]$^-$ in MSJL extract.

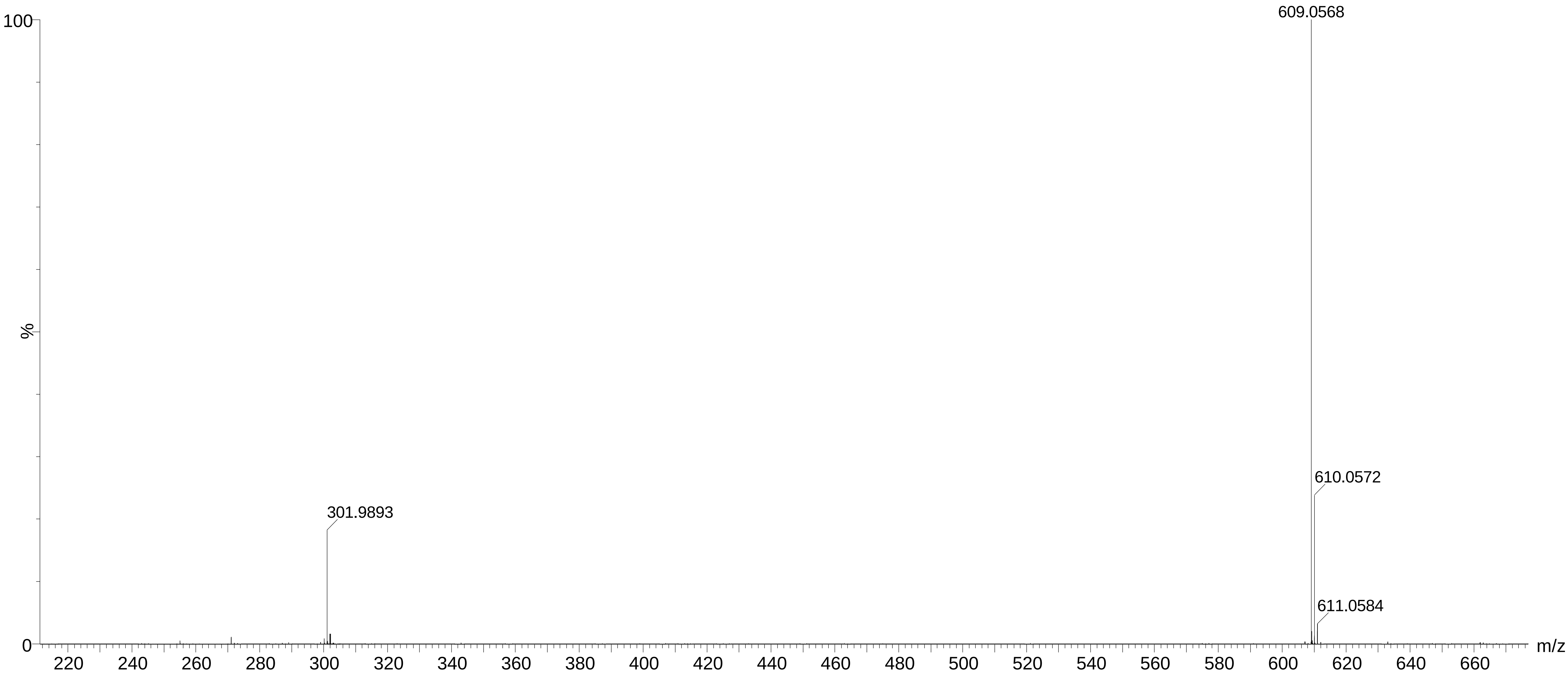

Fig. S29. MS^2 spectrum of 623.1573 *m/z* [M-H]$^-$ in MSJL extract.

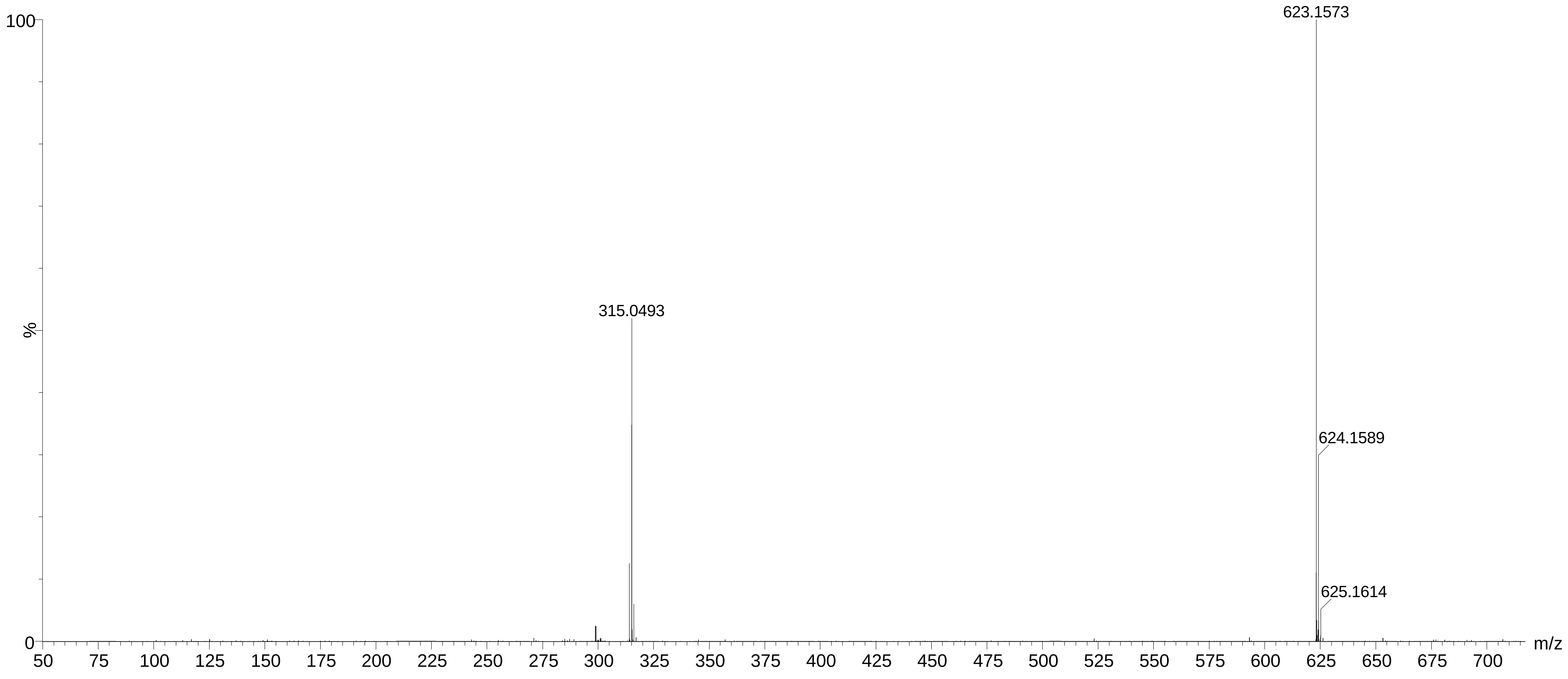

Fig. S30. MS^2 spectrum of 329.0812 *m/z* [M-H]$^-$ in MSJL extract.

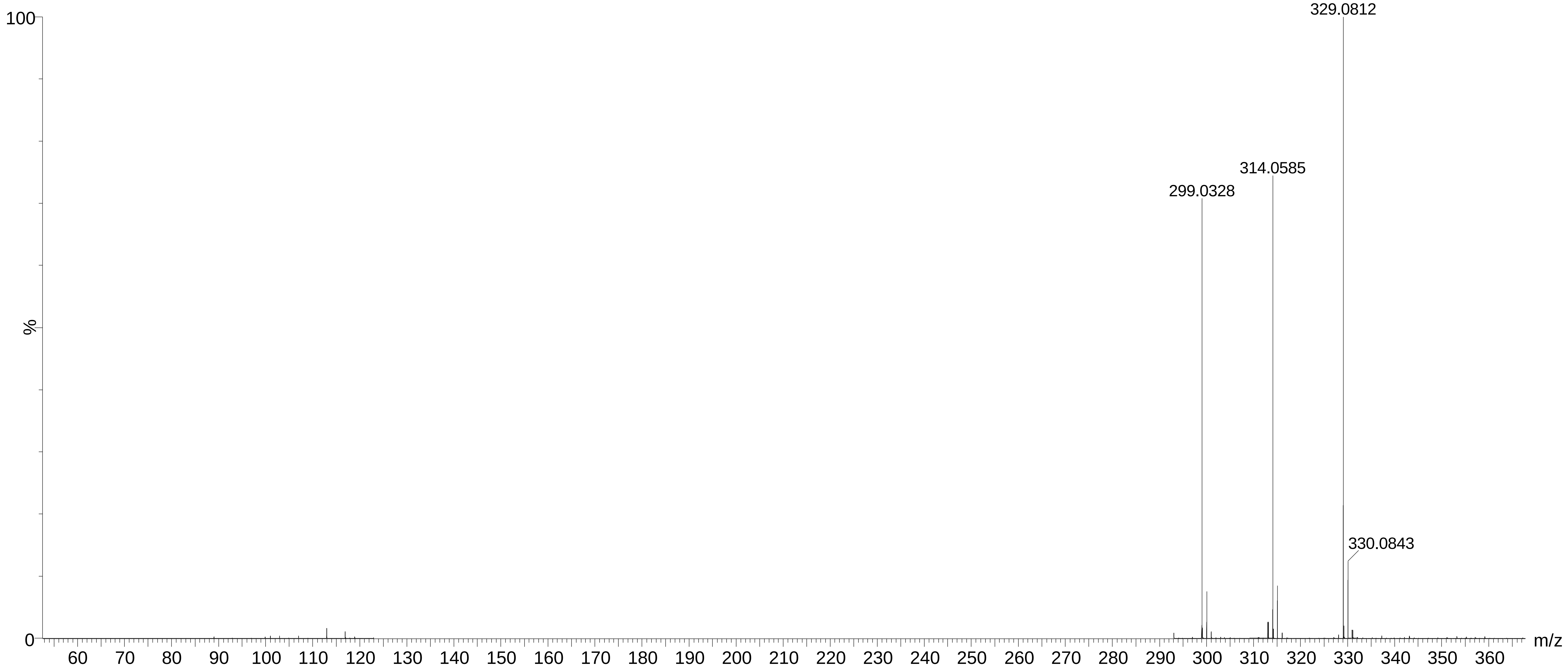

Fig. S31. MS^2 spectrum of 633.5037 *m/z* [M-H]$^-$ in MSJL extract.

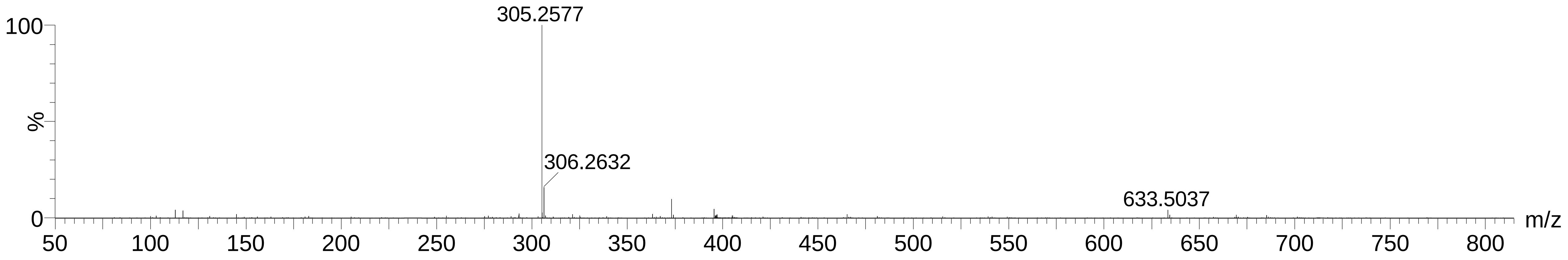

Supplement: Supplementary file 1 [file antibiotics-12-01601-s001.zip › 5. Supplementar material LCMS Jatoba 10 10 2023.pdf]
