# Supplementary material for: Evaluation of the Antioxidant, Antimicrobial, and Anti-Biofilm Effects of the Stem Bark, Leaf, and Seed Extracts from Hymenaea courbaril and Characterization by UPLC-ESI-QTOF-MS/MS Analysis
Source: Antibiotics (Basel). 2023 Nov 8;12(11):1601. doi: 10.3390/antibiotics12111601 (PMC10668761; doi:10.3390/antibiotics12111601)
Supplement: Supplementary file 1 [file antibiotics-12-01601-s001.zip › 6. Table of Abreviations 10 10 2023.pdf]

Abbreviations	Description
WHO	World Health Organization
PT	Total Phenolics
GAE	Equivalent in gallic acid
SM	Static Maceration
UB	Ultrasonic Bath
MS	Magnetic Stirring
SMJB	Static Maceration Jatobá Bark
UBJB	Ultrasonic Bath Jatobá Bark
MSJB	Magnetic Stirring Jatobá Bark
SMJL	Static Maceration Jatobá Leaf
UBJL	Ultrasonic Bath Jatobá Leaf
MSJL	Magnetic Stirring Jatobá Leaf
SMJS	Static Maceration Jatobá Seed
UBJS	Ultrasonic Bath Jatobá Seed
MSJS	Magnetic Stirring Jatobá Seed
BHTE	Butylated Hydroxytoluene Equivalents
ATCC	American Type Culture Collection
MIC	Minimal Inhibitory Concentration
MTT	Mono-tetrazolium
DMSO	Dimethyl Sulfoxide
UPLC-HRMS/MS	Ultra-high Performance Liquid Chromatography Coupled to High-Resolution Mass Spectrometry
EHE	Hydroethanolic Extract
UFU	Federal University of Uberlândia
HUFU	Herbarium of the Federal University of Uberlândia
TSA	Tryptone Soy Agar
TSB	Tryptone Soy Broth
CFU	Colony Forming Unit
MHA	Muller-Hinton Broth
ESI	Electrospray Ionization
